# Synthesis and Preliminary Studies for In Vitro Biological Activity of Two New Water-Soluble Bis(thio)carbohydrazones and Their Copper(II) and Zinc(II) Complexes

**DOI:** 10.3390/ijms251910831

**Published:** 2024-10-09

**Authors:** Alessio Zavaroni, Elena Riva, Valentina Borghesani, Greta Donati, Federica Santoro, Vincenzo Maria D’Amore, Matteo Tegoni, Giorgio Pelosi, Annamaria Buschini, Dominga Rogolino, Mauro Carcelli

**Affiliations:** 1 Department of Chemistry, Life Sciences and Environmental Sustainability, University of Parma, Parco Area delle Scienze 17/A, 43124 Parma, Italy; alessio.zavaroni@unipr.it (A.Z.); elena.riva@unipr.it (E.R.); valentina.borghesani@unipr.it (V.B.); matteo.tegoni@unipr.it (M.T.); giorgio.pelosi@unipr.it (G.P.); annamaria.buschini@unipr.it (A.B.); dominga.rogolino@unipr.it (D.R.); 2 Department of Pharmacy, University of Naples Federico II, Via Domenico Montesano, 49, 80131 Napoli, Italy; greta.donati@unina.it (G.D.); federica.santoro@unina.it (F.S.); vincenzomaria.damore@unina.it (V.M.D.); 3 Centre for Molecular and Translational Oncology, University of Parma, Parco Area delle Scienze 11/A, 43124 Parma, Italy

**Keywords:** thiocarbohydrazones, metallodrugs, metal complexes, cytotoxic activity, copper(II) complexes

## Abstract

Research in the field of metallodrugs is continually increasing. However, it is often limited by the poor solubility in water of the metal complexes. To try to overcome this problem, the two new ligands bis-(sodium 3-methoxy-5-sulfonate-salicylaldehyde)thiocarbohydrazone (**bis-TCH**, Na_2_H_4_L^1^) and bis-(sodium 3-methoxy-5-sulfonate-salicylaldehyde)carbohydrazone (**bis-CH**, Na_2_H_4_L^2^) were synthesized and characterized, both achieving high solubility in water. The speciation of the ligands and their coordinating behaviour towards the biologically relevant Cu(II) and Zn(II) ions were studied spectroscopically and potentiometrically, determining the p*K*_a_s of the ligands and the formation constants of the complex species. The monometallic and bimetallic Cu(II) and Zn(II) complexes were isolated, and the single-crystal X-ray structure of [Cu_2_(NaHL^1^)(H_2_O)_7_]^.^3.5H_2_O was discussed. Finally, preliminary studies of the in vitro cytotoxic properties of the new compounds were started on normal (Hs27) and cancer (U937) cell lines. **bis-TCH** was able to induce a growth inhibition effect between 40% and 45% in both cell lines; **bis-CH** did not produce a reduction in cell viability in Hs27 cells but revealed mild antiproliferative activity after 72 h of treatment in U937 cancer cells (GI_50_ = 46.5 ± 4.94 μg/mL). Coordination of the Cu(II) ions increased the toxicity of the compounds, while, in contrast, Zn(II) complexes were not cytotoxic.

## 1. Introduction

**Scheme 1:**
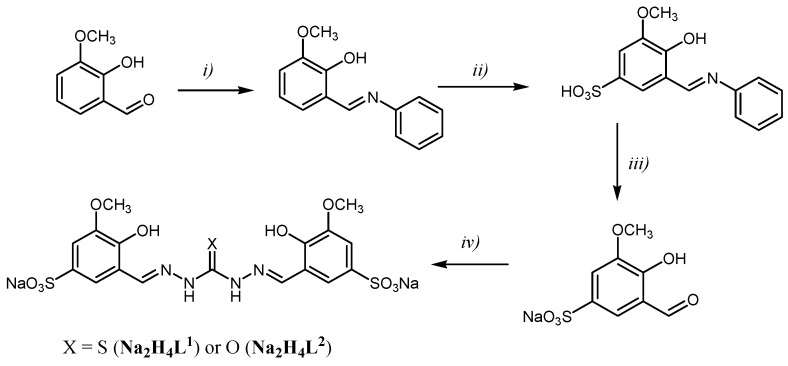
Synthetic pathway to obtain the ligands **bis-TCH** (Na_2_H_4_L^1^) and **bis-CH** (Na_2_H_4_L^2^). Reagents and conditions: (**i**) aniline, ethanol, reflux, 2 h; (**ii**) concentrated sulfuric acid, 105 °C, 2 h; (**iii**) Na_2_CO_3_, H_2_O, 115 °C, 2 h; then, CH_3_COOH until pH = 5; (**iv**) thiocarbohydrazide or carbohydrazide, H_2_O/ethanol 1/4, reflux, 1 h 30′.

The scientific literature regarding thiosemicarbazones and bis-thiosemicarbazones has developed enormously over the last fifty years [1]. It is well established that thiosemicarbazones can intervene, as ligands or as complexes, in the homeostasis of iron and copper, provoking programmed cell death [2,3,4]. In 2015–2016, there was a decisive turning point in these studies with the identification of COTI-2 (developed by Critical Outcome Technologies Inc., London, ON, Canada) and DpC (di-2-pyridylketone 4-cyclohexyl-4-methyl-3-thiosemicarbazone, developed by Oncochel Therapeutics), which have nanomolar cytotoxic activity and are now in phase I clinical development [5]. The activity of these thiosemicarbazones does not seem attributable to the traditionally invoked molecular mechanisms, i.e., chelation of iron ions and production of ROS, or inhibition of the enzyme ribonucleotide reductase. Instead, chelation of Cu(II) ions (and perhaps Zn(II) as well) and proteins such as p53 and protein disulfide isomerase have emerged as alternative molecular targets, within a multifactorial framework [6,7]. The coordination chemistry of bis-thiocarbohydrazone ligands and the biological properties of their metal complexes have been much less investigated [8,9,10]. The study of such ligands and of their metal complexes is often hampered by their poor solubility in water [8]. We have recently tried to overcome this problem by synthesizing sulfonated thiosemicarbazone derivatives; these have actually shown excellent solubility in water at physiological pH [11]. In this work, we tried to apply the same strategy, and the synthesis and characterization of the sulfonated bis-thiocarbohydrazone **bis-TCH** and bis-carbohydrazone **bis-CH** are discussed (Scheme 1). First, we performed some studies in solution to characterize the behaviour of these two ligands and to study their complexation with the biologically relevant Cu(II) and Zn(II) ions. Bis-(thio)carbohydrazones are able to coordinate one or two metal ions [12], depending on the reaction conditions. We have therefore studied the possibility of obtaining monometallic (**C1**–**C4**) and bimetallic (**C5**–**C8**) complexes of Cu(II) and Zn(II). A preliminary study of the in vitro cytotoxic properties of the ligands and of the complexes **C1**–**C4** was started, evaluating the antiproliferative activity on normal (Hs27) and cancer (U937) cell lines. The GI_50_ value was evaluated at different times (24, 48, and 72 h) and treatment concentrations (0–100 µg/mL), and in the discussion of the data, we begin to outline possible structure–activity correlations.

## 2. Results and Discussion

### 2.1. Synthesis

The two novel ligands **bis-TCH** and **bis-CH** were synthesized as outlined in Scheme 1. 2-hydroxy-3-methoxybenzaldehyde was sulfonated in position 5 following procedures described in the literature [13], and the product was condensed with thiocarbohydrazide or carbohydrazide in a 2:1 molar ratio to give the sodium salts of the ligands Na_2_H_4_L^1^ 5.5 H_2_O (**bis-TCH**) and Na_2_H_4_L^2^∙5H_2_O (**bis-CH**) at high yields (Scheme 1).

The IR spectrum of **bis-TCH** (Figure S1) is characterized by two absorption bands at 1647 and 1612 cm^−1^, related to the stretching of the C=N bonds; the absence of the S-H stretching band around 2600 cm^−1^ indicates that, in the solid state, the ligand is in the thione form. The IR spectrum of **bis-CH** displays a band at 1663 cm^−1^ (stretching of the C=O group) and two bands at 1614 and 1586 cm^−1^, attributable to C=N stretching (Figure S2).

In the ^1^H NMR spectrum of **bis-TCH** in d_6_-DMSO at 298 K (Figure S3A), there are no signals attributable to the SH group (typically at ca. 15 ppm), suggesting that, in solution as well, the ligand exists as a thione tautomer, as also confirmed by the signal at about 174 ppm in the ^13^C NMR spectrum (Figure S3B). The ligands could be present in solution as different configurational isomers around the two imine bonds, namely EE, EZ, and ZZ; in the case of **bis-TCH**, the pattern of signals observed is consistent with the presence of the unsymmetrical EZ form, while the ^1^H NMR spectrum of **bis-CH** shows a pattern of peaks attributable to a symmetrical configuration (Figures S3 and S4).

The solubility of the ligands in water at r.t. is approximately 10 g/L and 23 g/L for **bis-TCH** and **bis-CH**, respectively. Elemental (see experimental section) and TGA (Figure S5) analyses confirmed the formulae Na_2_H_4_L^1^∙5.5H_2_O (**bis-TCH**) and Na_2_H_4_L^2^∙5H_2_O (**bis-CH**).

The ligands were reacted with copper(II) or zinc(II) ions at a 1:1 or 1:2 ligand/metal ratio, leading to the formation of the complexes **C1**–**C8** (Table 1).

All the metal complexes were characterized by FT-IR spectroscopy, elemental analysis (see experimental section), ESI mass spectrometry, and, in the case of the zinc(II) complexes, ^1^H NMR as well (Figures S6–S14).

The complexes C1 and C2 were prepared by reacting CuCl_2_ with **bis-TCH** and **bis-CH**, respectively, at a 1:1 metal/ligand ratio in H_2_O/MeOH at room temperature. The involvement of the imine nitrogen atoms in copper coordination is confirmed by the IR spectra: the bands related to C=N stretching of the free ligands shift to lower frequencies and gain intensity in **C1** (from 1647, 1612 cm^−1^ to 1607, 1586 cm^−1^) and **C2** (from 1614, 1586 cm^−1^ to 1604, 1558 cm^−1^). In the IR spectrum of **C2,** the C=O stretching band has a moderate shift to lower values (from 1663 in the ligand to 1651 cm^−1^, Δν = 12 cm^−1^), confirming the participation of the keto oxygen in the metal ion coordination (Figure S15). IR data and elemental analysis suggested that **C1** and **C2** have the common formula CuNaH_3_L∙nH_2_O, with the salicylic oxygen deprotonated and the ligands behaving as ONX tridentate (X=S, n = 4; X=O, n = 5). The zinc(II) complexes **C3** and **C4** were prepared by reacting Zn(CH_3_COO)_2_ with the two ligands in a 1:1 metal/ligand ratio. As for the copper complexes, the shift of the C=N stretching vibrations to lower frequencies indicates the involvement of the imine nitrogen atoms in zinc coordination. In the IR spectrum of **C4**, the C=O band is now significantly shifted to lower frequencies with respect to the same band of **bis-CH** (Δν = 63 cm^−1^, Figure S16). In this case and in **C3**, the ligand seems bi-deprotonated (salicylic and hydrazidic hydrogens) and coordinates to the metal ion in the enol form in a tridentate ONO fashion. Elemental analyses made it possible to assign the general formula ZnNa_2_H_2_L∙n iPrOH∙mH_2_O for **C3** and **C4**.

The copper(II) complexes **C5** and **C6** were prepared by reacting CuCl_2_ respectively with **bis-TCH** and **bis-CH**, respectively, in a 2:1 metal/ligand ratio. In the IR spectrum of **C6**, the band of C=O stretching falls at 1596 cm^−1^, indicating that the **bis-CH** is coordinated in the enol form (Figure S15). The elemental analyses suggested that the two complexes have the formula Cu_2_NaHL∙nH_2_O, as also supported by ESI mass spectrometry (Figures S11 and S12) and X-ray diffraction analysis of [Cu_2_(NaHL^1^)(H_2_O)_7_]^.^3.5H_2_O (see Crystallography section).

The complexes **C7** and **C8** were prepared by reacting Zn(CH_3_COO)_2_ with **bis-TCH** and **bis-CH**, respectively, in a 2:1 metal-to-ligand ratio. Even for **C8,** as with **C4** and **C6**, the IR spectrum is characterized by a C=O stretching band at lower frequencies than in the free ligand (1595 cm^−1^, Figure S16). In the ^1^H NMR spectra, the peak associated to the methyl protons of the acetate group is present (see Figure S6 as an example). These results, together with elemental analysis, led us to propose Zn_2_(Na_2_HL)(CH_3_COO)·nH_2_O as a general formula for **C7** and **C8**. It is relevant to note that the characterization of the complexes **C5**–**C8** is complicated by their hygroscopic nature.

### 2.2. Proton Dissociation Processes

Proton dissociation constants provide information on the capacity of a ligand to form complexes at different pH values. Indeed, ligands that contain strongly acidic groups will have a tendency to interact with metal ions at a lower pH than ligands whose functional groups have higher p*K_a_* values. Moreover, knowledge of the protonation constants of the acidic groups on a molecule may provide an opportunity for greater comprehension of its biological activity. In fact, the total charge of the molecule (either as a free ligand or in the form of a metal complex) can strongly influence its capacity to interact with biomolecules and cell membranes.

**Scheme 2:**
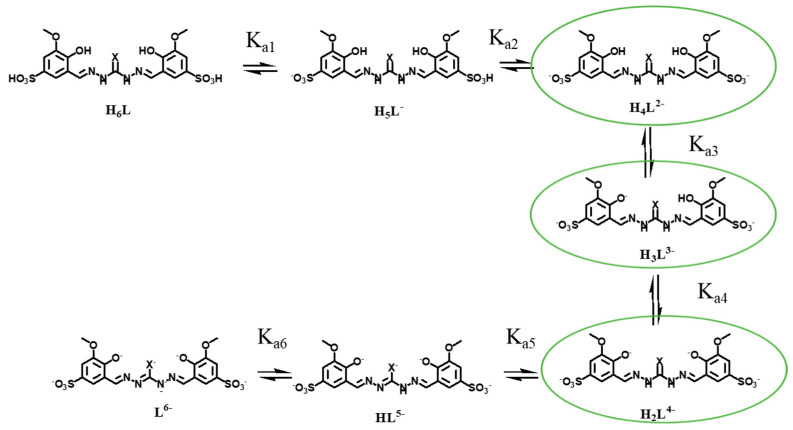
Proton-dissociation equilibria for the two ligands (X = S, O); species in aqueous solution in the pH range 4–10 are shown in green circles.

In their fully protonated state, the two ligands can be regarded as hexa-protic acids (H_6_L, Scheme 2); however, in aqueous solution, they behave as triprotic acids in the pH range 2–12.

**Figure 1:**
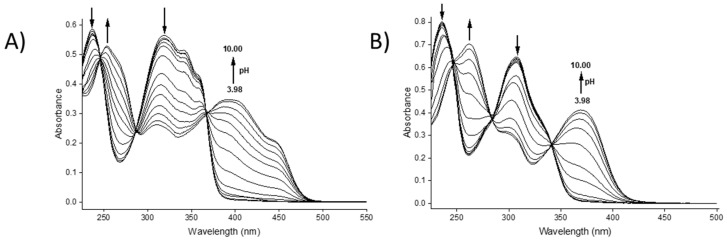
UV–visible absorption spectra for (**A**) **bis-TCH** and (**B**) **bis-CH** in aqueous solution at different pH values (pH range: 3.98–10.00; *T* = 298.2 K, C_L_ = 17 µM).

The proton dissociation constants of Na_2_H_4_L^1^·5.5 H_2_O (**bis-TCH**) were studied by registering the UV–visible spectra in aqueous solution between pH 3.98 and 10.00 (Figure 1A).

**Figure 2:**
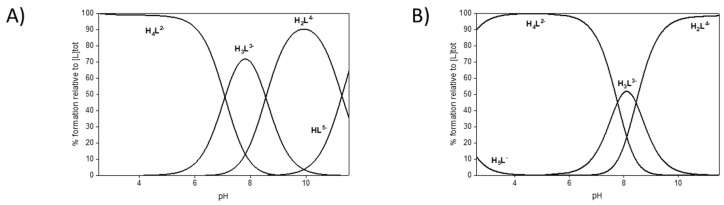
Representative distribution diagram of (**A**) **bis-TCH** and (**B**) **bis-CH** in aqueous solution (C_L_ = 3 mM, *I* = 0.1 M KCl, *T* = 298.2 K). For each pH value (x axis), the amount of ligand in its different protonation states is reported as a % of the total ligand in solution. At physiological pH, the major species are H_4_L^2−^ and H_3_L^3−^. The former correspond to the ligand deprotonated on the sulfonic groups, while the latter to the ligand deprotonated on the two sulfonic groups and one phenolic functional group.

Between pH = 3.98 and 6.00, the solutions were colourless with absorption maxima at 237 and 320 nm. From pH = 6.49, the solution was pale green, and the colour became more intense until pH = 10.00; the colour change was accompanied by the appearance of bands at 253 and 393 nm, with a shoulder at 445 nm. There are three apparent isosbestic points at 246, 288, and 368 nm, but a deeper analysis revealed that they shift during the titration, suggesting the presence of three species rather than two and therefore two dissociation equilibria (Figure S17) [14]. Indeed, spectroscopic data processing with HypSpec2014 software allowed us to determine two p*K_a_* values (Table 2) that corresponded to the dissociation of the two phenolic groups (p*K_a_*_3_ = 6.78(1) and p*K_a_*_4_ = 8.77(1), Scheme 2) [15]. Therefore, the single-site p*K_a_* value of the phenolic groups can be estimated as the average p*K_a_*, which is 7.78. This value is similar to that reported for the deprotonation of the phenolic moiety of sodium 5-sulfonate-salicylaldehyde thiosemicarbazones [16]. The values of p*K_a_*_1_ and p*K_a_*_2_, associated with the proton dissociation of the sulfonic groups, are expected to have much lower values (below 3) and could not be determined from these experiments. Potentiometric titrations were performed in an attempt to determine the value of the p*K_a_* of the hydrazidic NH protons (p*K_a_*_5_ and p*K_a_*_6_) and, possibly, those of the protonated sulfonate groups. Effectively, potentiometric data allowed us to determine the p*K_a_*_5_ value, along with p*K_a_*_3_ and p*K_a_*_4_ (Table 2). The values of p*K_a_*_3_ and p*K_a_*_4_ obtained by potentiometry are consistent with those obtained by spectrophotometric titrations. A representation of the distribution diagram for **bis-TCH** is reported in Figure 2A.

Similarly, the proton dissociation constants of **bis-CH** were studied spectrophotometrically in aqueous solution between pH = 3.98 and 10.00 (Figure 1B). At pH values between 3.98 and 6.49, the spectrum of the ligand did not change, and two strong bands at 235 and 308 nm were observed. A significant spectral change was observed in the pH range 7.00–10.00 (Figure 1B). As for **bis-TCH**, the isosbestic points at 247, 284, and 341 nm (Figure S18) shift in the course of the titration, suggesting the presence of three species in this pH range and therefore two dissociation equilibria. The spectrophotometric data were processed by means of HypSpec2014 software, leading to p*K_a_*_3_ = 7.34(1) and p*K_a_*_4_ = 8.58(1), which were assigned to the deprotonation of the two phenolic OH groups (Table 2). As for **bis-TCH**, the single-site p*K_a_* value of the phenolic groups can be estimated as the average p*K_a_* (7.96). Additionally, to complete the analysis, we carried out potentiometric titrations of **bis-CH** starting from a very acidic pH. Indeed, starting from pH = 2.60, we were able to observe three dissociation equilibria, with p*K_a_* values of 1.70(10), 7.77(3), and 8.45(3) (Table 2). The values obtained by potentiometry are consistent with those obtained by spectrophotometric titrations (Table 2), and the lowest of the p*K_a_* values was associated with the proton dissociation of H_5_L^−^ on the second sulfonic group (p*K_a_*_2_ = 1.70). Importantly, no dissociation of the hydrazidic NH groups was observed, a behaviour consistent with that reported in the literature [17]. A representative distribution diagram of **bis-CH** is reported in Figure 2B.

### 2.3. Cu(II)/bis-TCH Complexation Equilibria

**Figure 3:**
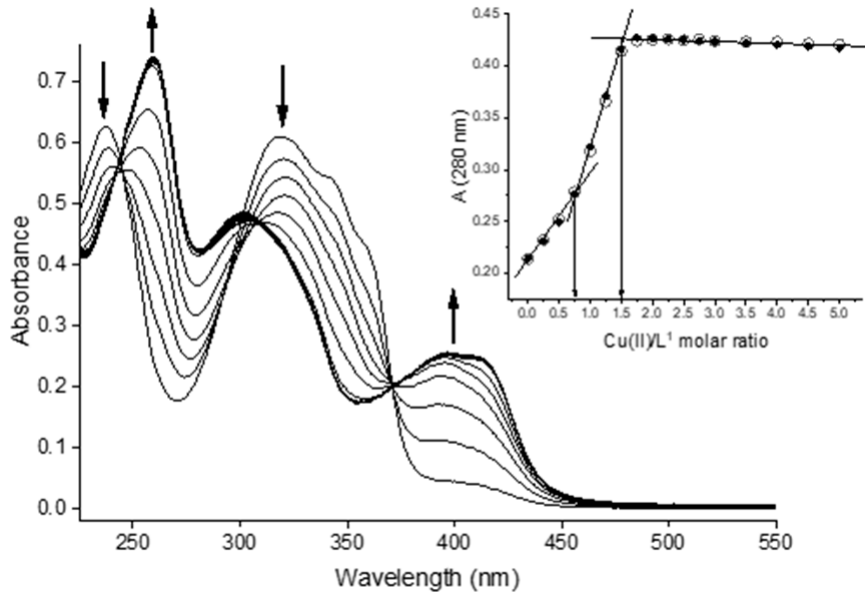
UV–visible spectra for the titration of **bis-TCH** with Cu(II) in aqueous solution at pH 2.5 (C_L_ = 17 µM; Cu(II):L = 0–5). Inset: plot of the observed (circles) and calculated (dots) absorbance values at 280 nm vs. Cu(II)/ligand molar ratio.

The copper(II) complexation equilibria of **bis-TCH** were studied by means of spectrophotometric titration of the ligand with Cu(II) in aqueous solution at pH = 2.5 and pH = 7.4. It is worth noting that these titrations, being performed at constant pH, did not allow us to establish the protonation state of the ligand but only the possible stoichiometry (Cu/L) of the species. For this reason, in the speciation models, L identifies the ligand irrespective of its protonation state, and the charges of the complexes are omitted. Spectral data at pH = 2.5 were collected by titration of **bis-TCH** (C_L_ ca. 17 µM) with Cu^2+^ ions up to a metal/ligand ratio of 5 (Figure 3). The two bands at 237 and 318 nm, observed in the spectrum of the free ligand, decrease in absorbance upon addition of Cu(II), while the two bands at 259 and 400 nm increase. A thorough examination of the titration data showed that three species are present in solution: the ligand and two complex species.

A plot of absorbance at 280 nm vs. Cu(II)/ligand molar ratio (Figure 3, inset) quite clearly evidenced that two equivalence points are present: one at ca. 0.75 eq. and one at ca. 1.5 eq. of metal added, corresponding to the formation of [Cu_3_L_4_] and [Cu_3_L_2_] species. Data treatment with these two species in the model resulted in the fitting of the curve and the determination of the log *β* values (Table 3); the complex species have remarkable stability and are already formed at a low pH. Note that a model with [CuL] and [Cu_2_L] does not allow fitting of the data in a manner consistent with the observed Cu/L ratios at the titration end points, as described above. The [Cu_3_L_4_] species could have a simple structural explanation considering some peculiar characters of **bis-TCH**. This ligand, in fact, adds to the coordinating possibilities of the central skeleton (ONSNO) two sulfonic groups, which are deprotonated, such that they can also behave as counteranions. Moreover, both the ligands have a great affinity for Cu(II) ions, but **bis-TCH** is more acidic than **bis-CH**, and this implies that it more stably coordinates Cu(II) already at pH 2.5. It will be seen further in the discussion that in the case of **bis-CH** at pH = 2.5, the [CuL] species is identified, while, at the same pH, **bis-TCH** is able to compete more efficiently for the Cu(II) ions in solution, and the result is [Cu_3_L_4_], a species with a higher ligand/metal ratio (Figure S19).

**Figure 4:**
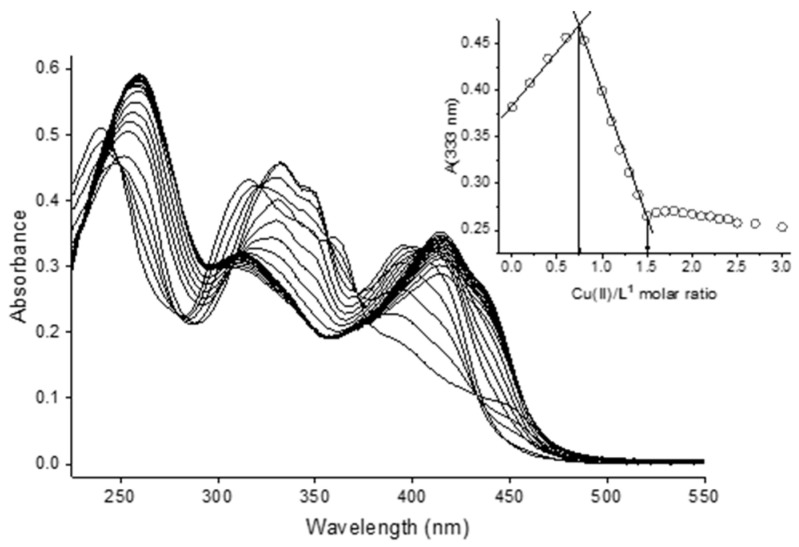
UV–visible spectra for the titration of **bis-TCH** with Cu(II) in aqueous solution at pH 7.4 (25 mM HEPES buffer; C_L_ = 17 µM; Cu(II):L = 0–3). Inset: plot of the observed absorbance values at 333 nm vs. Cu(II)/ligand molar ratio.

Titration spectral data were collected on a solution of **bis-TCH** (C_L_ ca. 17 µM) in aqueous 25 mM HEPES buffer at pH 7.4 titrated with Cu^2+^ ions up to a metal/ligand ratio of 3. Figure 4 reports the entire spectral set of the titration, together with the plot of the absorbance values at 333 nm with respect to the equivalents of Cu(II) added to the ligand solution. With the addition of copper(II), the light green colour of the solution turned darker. There are two equivalence points, at ca. 0.75 and 1.5 eq. of Cu(II) added, which are consistent with the formation of [Cu_3_L_4_] and [Cu_3_L_2_] species, as observed at pH 2.5.

The analysis revealed the presence of three families of spectra. The first comprises the spectra collected after addition of Cu(II) between 0 and 0.8 equivalents with respect to the ligand (Figure S20): we can note the presence of at least six isosbestic points at 250, 280, 321, 357, 371, and 433 nm. We can see the decrease in the intensity of the bands at 240, 316, 361, and 448 nm; the formation of bands at 252, 333, and 346 nm; and the increase in the intensity of the band at 396 nm. The second group (Figure S21) comprises the spectra collected after the addition of Cu(II) between 0.8 and 1.5 equivalents. This family is characterized by the presence of three isosbestic points at 232, 310, and 405 nm. The band at 333 nm decreases in intensity, while there is an increase in the intensities of the bands at 261 and 415 nm. The trend in the absorbance after 1.5 eq. of metal cannot be accounted for by simple dilution (e.g., the decrease in absorbance at 413 nm after 1.5 eq. of Cu(II) added, as shown in Figure S22). Therefore, a third family of spectra can be identified (Figure S23). These spectra are characterized by the small increase in the intensity of the band at 261 nm, the slight decrease in the intensity of the band at 415 nm, and the presence of one isosbestic point at 380 nm. As already stated, the change in absorbance after the addition of 1.5 eq. of Cu(II) was not accounted by a simple dilution effect; we made the hypothesis that, in the presence of excess copper(II), a species such as [Cu_2_L] can be formed at pH 7.4. Unfortunately, and contrarily to what was obtained from data collected at pH 2.5, we could not obtain a reasonable convergence in the log *β* values. Although a speciation model consisting of [Cu_3_L_4_], [Cu_3_L_2_], and [Cu_2_L] seems reasonable based on the spectral dataset, the steep inflection points in the absorbance values vs. metal added (Figure 4) suggest that, at pH 7.4, the formation constants are too high to be determined.

### 2.4. Cu(II)/bis-CH Complex Formation Equilibria

**Figure 5:**
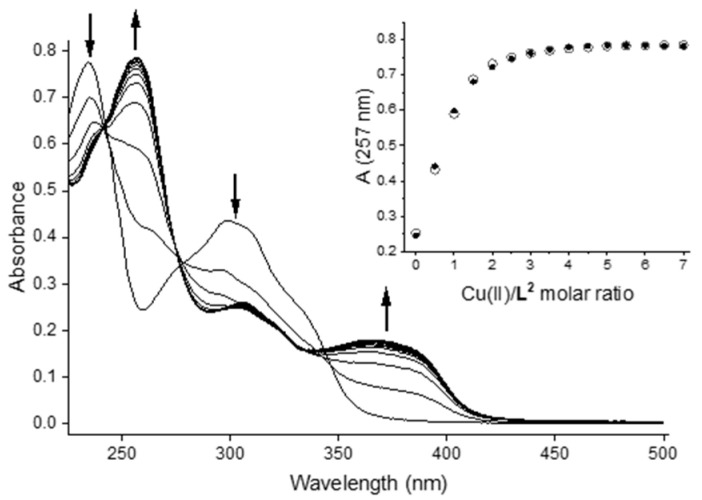
UV–visible spectra for the titration of **bis-CH** with Cu(II) in aqueous solution at pH 2.5 (C_L_ = 17 µM; Cu(II):L = 0–7). Inset: plot of the observed (circles) and calculated (dots) absorbance values at 257 nm vs. Cu(II)/ligand molar ratio.

The copper(II) complexation equilibria of **bis-CH** were studied by means of spectrophotometric titration at pH = 2.5 and 7.4. Again, it is worth noting that titrations conducted at constant pH did not allow us to establish the protonation state of the ligand, which is simply reported as L irrespective of its protonation state, but only the possible stoichiometry (Cu/L) of the species. Spectral data at pH = 2.5 were collected by titration of **bis-CH** (C_L_ ca. 17 µM) in acidic solution with Cu^2+^ ions up to a metal/ligand ratio of 7 (Figure 5). As also observed with **bis-TCH**, significant changes in the spectra of the ligand upon addition of copper(II) show that complexes are formed at this low pH as well. The best fit of the spectrophotometric data was obtained by considering the formation of [CuL] and [Cu_2_L] species, with log *β* values of 6.26(2) and 11.30(2), respectively (Table 2). A representative speciation diagram is reported in Figure S24.

**Figure 6:**
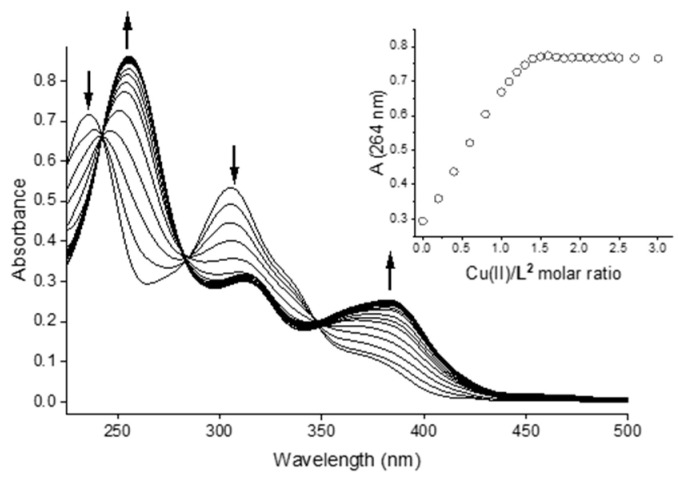
UV–visible spectra for the titration of **bis-CH** with Cu(II) in aqueous solution at pH 7.4 (25 mM HEPES buffer; C_L_ = 17 µM; Cu(II):L = 0–3). Inset: plot of the observed absorbance values at 264 nm vs. Cu(II)/ligand molar ratio.

Spectral data were collected by titration of a solution of **bis-CH** (C_L_ ca. 17 µM) in aqueous 25 mM HEPES buffer at pH 7.4 with Cu^2+^ ions up to a metal/ligand ratio of 3. In contrast to the data at pH 2.5, these spectral data reveal a steep equivalence point at 1.5 eq. of Cu vs. L (Figure 6). This behaviour corresponds to the formation of a predominant complex species of stoichiometry Cu/L 3:2. As for the **bis-TCH**, the presence of this pronounced titration endpoint did not allow us to calculate a conditional formation constant for a hypothetical [Cu_3_L_2_] species.

### 2.5. Zn(II)/bis-TCH and Zn(II)/bis-CH Complex Formation Equilibria

**Figure 7:**
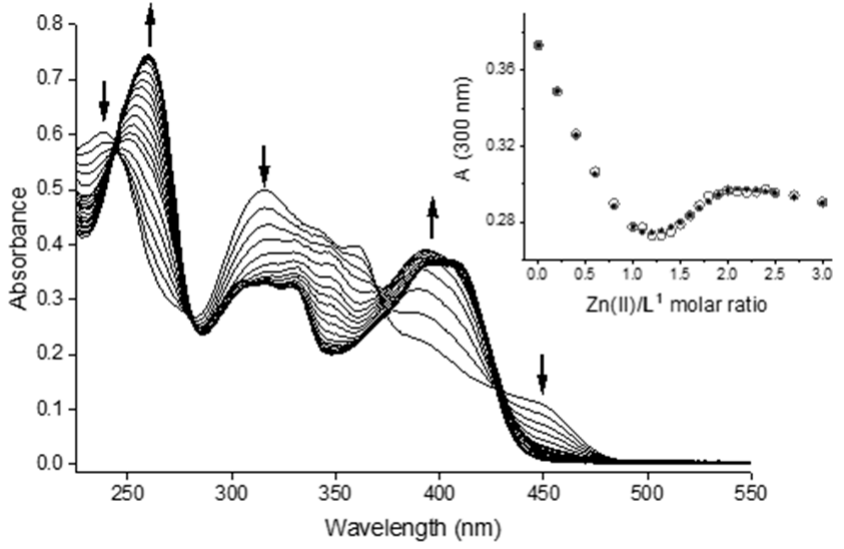
UV–visible spectra for the titration of **bis-TCH** with Zn(II) in aqueous solution at pH 7.4 (25 mM HEPES buffer; C_L_ = 17 µM; Zn(II):L = 0–3). Inset: plot of the observed (circles) and calculated (dots) absorbance values at 300 nm vs. Zn(II)/ligand molar ratio.

A solution of Na_2_H_4_L^1^·5.5 H_2_O (C_L_ ca. 17 µM) in aqueous 25 mM HEPES buffer at pH 7.4 was titrated with Zn^2+^ ions up to a metal/ligand ratio of 3 (Figure 7).

The best fit was obtained using a speciation model that consists of the sequential formation of a monometallic [ZnL] and a bimetallic [Zn_2_L] species. In fact, two families of spectra were observed, according to the presence in solution of two complex species and the free ligand. The first family of spectra (Zn/L = 0–1) (Figure S25) is characterized by a decrease in intensity for the bands of the free ligand at 239, 315, and 450 nm, and the appearance of bands at 251 and 394 nm. There are four isosbestic points at 245, 280, 371, and 429 nm. The second family of spectra (Zn/L = 1–2) (Figure S26) presents redshift and increase in absorbance of the band at 251 nm, the disappearance of the band at 349 nm, and redshift with decrease of absorbance of the band at 394 nm. A plot of the absorbance values at 300 nm clearly shows the two equivalence points at 1 and 2 eq. of metal vs. ligand, consistent with the formation of [ZnL] and [Zn_2_L] species (Figure 7). The log *β* values for the formation of Zn(II) complexes with Na_2_H_4_L^1^ · 5.5 H_2_O can be estimated (Table 4), and a representative distribution diagram is reported in Figure S27.

**Figure 8:**
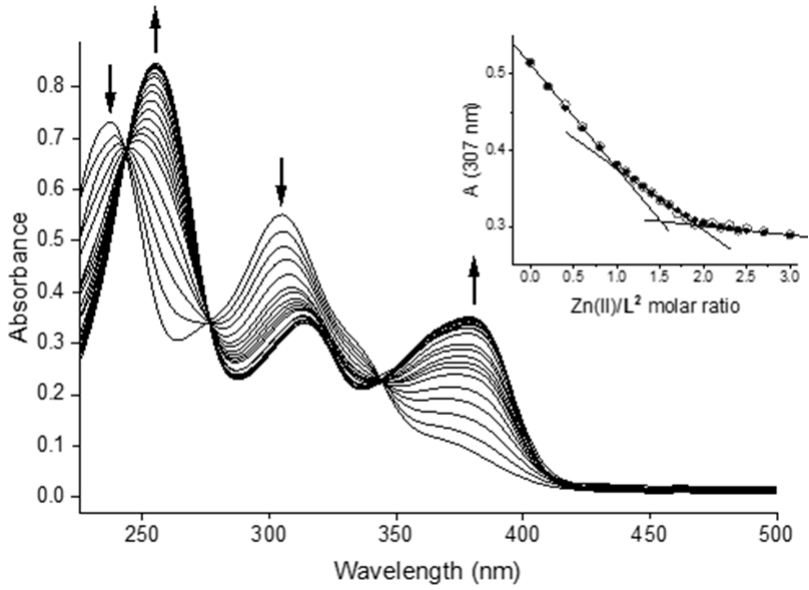
UV–visible spectra for the titration of **bis-CH** with Zn(II) in aqueous solution at pH 7.4 (25 mM HEPES buffer; C_L_ = 17 µM; Zn(II):L = 0–3). Inset: plot of the observed (circles) and calculated (dots) absorbance values at 307 nm vs. Zn(II)/ligand molar ratio.

The same study was undertaken with Na_2_H_4_L^2^ · 5 H_2_O: titration of the ligand (C_L_ ca. 17 µM) in aqueous 25 mM HEPES buffer at pH 7.4 with Zn^2+^ ions up to a metal/ligand ratio of 3 (Figure 8).

The spectra are characterized by four bands at ca. 238, 255, 305, and 371 nm, with those at 255 and 371 nm increasing in intensity with the addition of metal ions. The presence of these separated absorption bands led to the observation of three apparent isosbestic points at 243, 276, and 345 nm. Analogously to the Zn(II)/bis-thiocarbohydrazone system, the best fit was obtained using a speciation model that consists of the sequential formation of a monometallic [ZnL] and a bimetallic [Zn_2_L] species. In Figure 8, the absorbance values at 307 nm as a function of the equivalents of added Zn(II) are reported. The absorbance trend presents two almost linear regions between 0 and 1 eq. of Zn(II) and between 1 and 2 eq., respectively. This is, in fact, consistent with the sequential formation of two species with Zn:L *ratios* of 1:1 and 2:1, respectively. The calculated log *β* values are reported in Table 4, and a representative distribution diagram is presented in Figure S28. Overall, the comparison of the log *β* values for the formation of Zn(II) complexes with Na_2_H_4_L^1^ · 5.5 H_2_O and Na_2_H_4_L^2^ · 5 H_2_O indicates that the former gives rise to more stable complexes (ca. 1.5 log units for both species). This behaviour is, perhaps, to be expected, taking into account the softer nature of the thiocarbohydrazonic group.

Overall, the stability of the Cu(II) complexes is higher than those of Zn(II); in fact, the conditional constants of the former ion are too high to be determined at pH 7.4. This is in line with the Irving–Williams series, which predicts higher stability constants for Cu(II) than for Zn(II). Interestingly, both metals have a thermodynamic preference for **bis-TCH** than for **bis-CH**; this is, perhaps, not surprising giving the intermediate to soft nature of the two studied metal ions.

### 2.6. Crystallography

**Figure 9:**
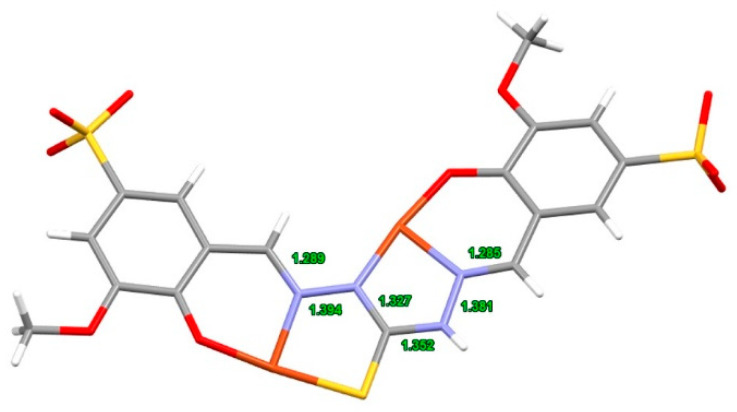
Crystal structure of the ligand arranged for chelation of the two Cu(II) ions in [Cu_2_(NaHL^1^)(H_2_O)_7_]^.^3.5H_2_O (oxygen atoms in red; sulphur in yellow; nitrogen in blue; carbon in grey and hydrogens in white; copper ions in orange).

Dark green crystals of [Cu_2_(NaHL^1^)(H_2_O)_7_]^.^3.5H_2_O were obtained from the mother solution of C1 synthesis. The crystal data reveal a peculiar structure in which the ligand presents two different situations around the thiocarbohydrazide moiety. The thiocarbohydrazide fragment shows a thiolic C-S single bond (1.725 Å) and a strongly delocalized π system over the NNCNN system. With respect to the two C-N bonds, the ligand presents an *E* configuration on one side and a *Z* configuration on the other side (Figure 9).

Such an atom disposition allows a double chelation: one with an ONS and the other with an NNO terdentate system. A further peculiarity is that the ligand is asymmetrically deprotonated and presents a total of five negative charges. In fact, one of the two hydrazidic nitrogen atoms that can potentially undergo deprotonation remains protonated, even upon chelation. These charges agree with the presence of a sodium ion, in addition to the two chelated Cu(II) ions, for a total of five positive charges.

**Figure 10:**
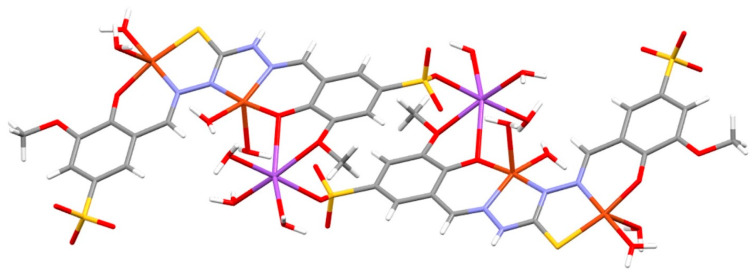
The bond of the sodium ion with the sulfonate of one ligand molecule and two oxygens from the salicylic moiety of another ligand molecule gives rise to dimers in the crystal structure of [Cu_2_(NaHL^1^)(H_2_O)_7_]^.^3.5H_2_O (oxygen atoms in red; sulphur in yellow; nitrogen in blue; carbon in grey and hydrogens in white; copper ions in orange; sodium ions in purple).

The coordination geometries of the two copper atoms are also remarkably different. The SNO-coordinated copper ion presents a more regular square planar coordination with the fourth position of the equatorial plane occupied by a water molecule, and a further apical water molecule is found to complete a square pyramidal geometry. This latter presents a very elongated Cu-O coordination bond (2.458 Å, Figure S29). The other copper atom presents a very distorted geometry that approaches a trigonal bipyramid in which the axial ligands are the three donor atoms from the thiocarbohydrazone (NNO), while the two remaining equatorial position are occupied by two water molecules (Figure S30). The sodium ion coordinates one sulfonate oxygen, two oxygens from the salicylic moiety, and three water molecules in a regular octahedral geometry (Figure S31). Curiously enough, the bond of sodium with the sulfonate creates in the crystal a head-to-tail system that gives rise to dimers (Figure 10).

### 2.7. Cytotoxicity against U937 and Hs27 Cell Lines

**Figure 11:**
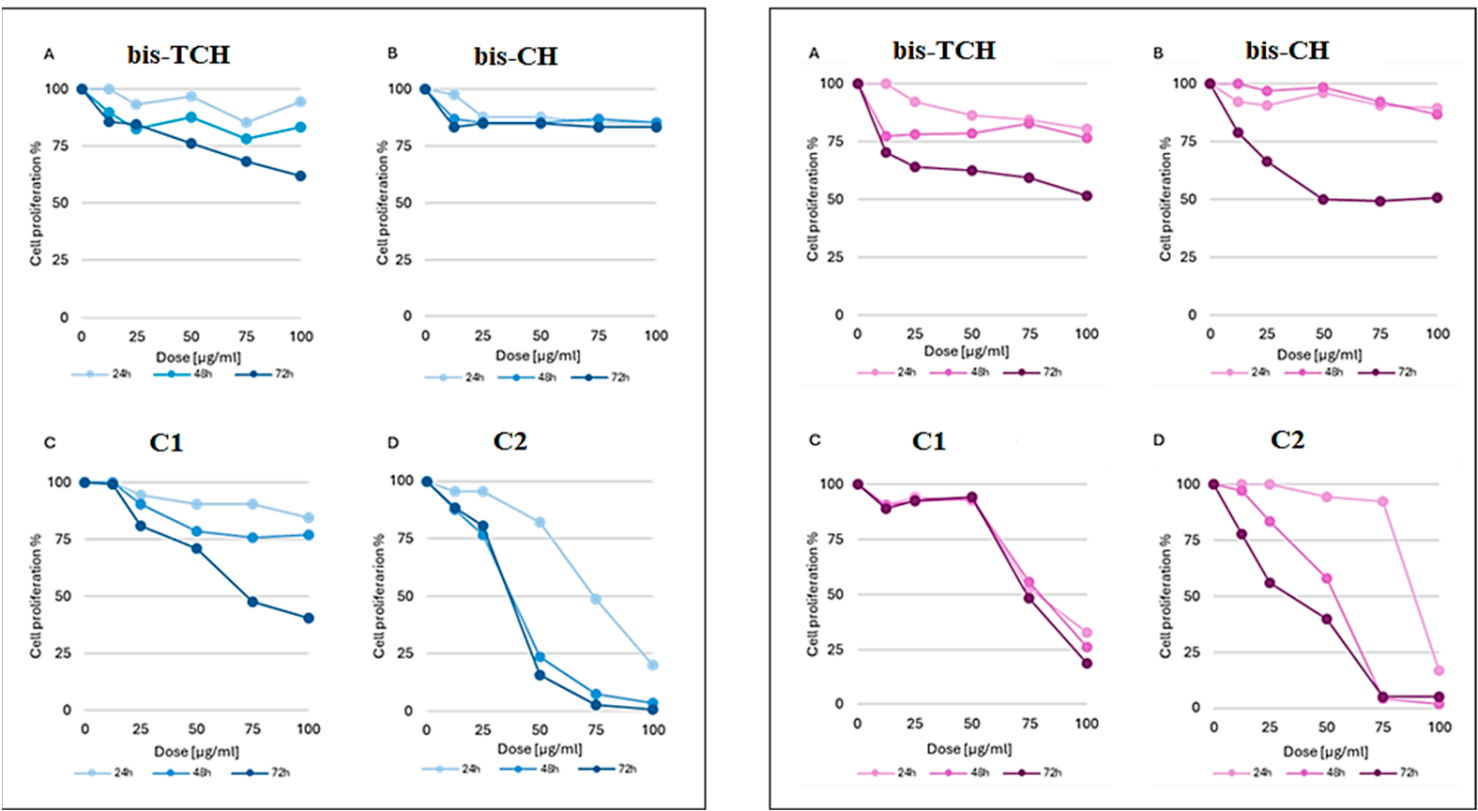
Dose–response curves obtained in Hs27 (**left**) and U937 cells (**right**) after 24, 48 and 72 h treatment with (**A**) **bis-TCH**; (**B**) **bis-CH**; (**C**) **C1**; (**D**) **C2**. Data are expressed as cell proliferation percentage compared with control cells.

We have started to investigate the antiproliferative profile of the ligands and of the complexes **C1**–**C4** using a normal fibroblast cell line (Hs27) and a leukaemia cell line (U937), an in vitro cell model system systematically used to validate the toxicological profile of new potential anticancer agents [18,19]. For the cytotoxicity assay, we treated Hs27 and U937 cells with five different concentrations of compound, ranging from 12.5 to 100 µg/mL, for 24, 48, and 72 h. The dose–response curves showed that **bis-TCH** was able to induce a growth inhibition effect between 40% and 45% in both cell lines in a dose- and time-dependent manner, and for the U937 cell line, it was able to cause a 50% reduction in cell viability only at the highest dose after 72 h of treatment (Figure 11).

**bis-CH** did not produce a reduction in cell viability in normal Hs27 cells but revealed mild antiproliferative activity after 72 h of application to U937 cancer cells (GI_50_ = 46.5 ± 4.94 μg/mL) (Figure 11). The coordination of Cu(II) ions increased the toxicity of the compounds. The dose–response curves of **C1** showed an important increase in cytotoxic effect on both cell lines. Particularly, in U937 cancer cells, a growth inhibition of 70% was achieved already at 24 h of treatment (Figure 11). The dose–response curves of **C2** showed a high reduction of proliferation, comparable in the two cell lines, that was also dose- and time-dependent (Figure 11). In contrast, Zn(II) coordination was not able to increase the cytotoxic activity of the two ligands (Figure S32). The GI_50_ values are reported in Table 5 (they are also reported in µM/L in Table S1).

## 3. Materials and Methods

Commercial reagents were purchased from Sigma-Aldrich. The purity of the synthesized compounds was determined by elemental analysis and verified to be ≥95%. ^1^H-NMR spectra were recorded at 25 °C on a Bruker Advance 400 MHz FT spectrometer. The ATR-IR spectra in the range of 4000–400 cm^−1^ were recorded by means of a Perkin Elmer spectrophotometer with a diamond crystal plate. Elemental analyses were performed by using a Thermo Fisher FlashSmart CHNS/O analyser with gas-chromatographic separation. Thermogravimetric analysis (TGA) was performed with a PerkinElmer TGA 8000 instrument (mass sample: 1–3 mg) under an atmosphere of air in the temperature range 30–300 °C, changing at 10 °C/min. Electrospray ionization mass spectral analysis (ESI-MS) was performed with an ESI time-of-flight Micromass 4LCZ spectrometer. Samples were dissolved in methanol. The UV–visible spectra were collected by means of a Thermo Scientific Evolution 260 Bio spectrophotometer equipped with a Peltier temperature controller, and quartz cuvettes with a 1 cm path length were used.

### 3.1. Synthesis

The ligands were synthesized by condensation of 3-methoxy-5-sulfonate-salicylaldehyde sodium salt with thiocarbohydrazide (**bis-TCH**) or carbohydrazide (**bis-CH**). 3-methoxy-5-sulfonate-salicylaldehyde sodium salt was obtained according to reported procedures [13], with slight modifications. 2-hydroxy-3-methoxybenzaldehyde (3.0 g, 0.02 mol) was dissolved in 40 mL of EtOH, and a solution of aniline (1.82 mL, 0.02 mol) in 4 mL of EtOH was added to the reaction mixture. The resulting brilliant red solution was refluxed for 2 h and cooled to room temperature, and the solvent was removed under vacuum. 2-methoxy-6-((phenylimino)methyl)phenol was isolated as an orange crystalline solid. Concentrated H_2_SO_4_ (d = 1.83 g/mL, 11.0 eq.) was then added, and the resulting mixture was stirred at 105 °C for 2 h. Ice water was slowly added to the mixture, and a yellow precipitate formed, which was filtered off, washed with cold water, and recrystallized in hot water. The beige precipitate was filtered off, washed with cold water, and dried under vacuum. To a suspension of the obtained 4-hydroxy-3-methoxy-5-((phenylimino)methyl)benzenesulfonic acid (3.108 g, 0.010 mol) in 7 mL of H_2_O, a solution of Na_2_CO_3_ (1.5 g, 0.014 mol, 1.4 eq.) in 7 mL of H_2_O was added dropwise. The resulting solution was stirred and boiled in an open flask for 2 h, then cooled to room temperature, and glacial acetic acid was added until the pH was ca. 5. Subsequently, EtOH (25 mL) was added to the solution, which was left at 4 °C overnight. A precipitate was obtained, which was filtered off, washed with EtOH, and dried under vacuum. Sodium 3-methoxy-5-sulfonate-salicylaldehyde was isolated as a brown–beige solid (1.674 g, Y = 65%). ^1^H NMR (400 MHz, d_6_-DMSO, 298K, ppm): δ = 3.85 (s, 3H; OCH_3_), 7.37 (d, J = 1.9 Hz, 1H; ArH); 7.52 (d, J = 1.9 Hz, 1H; ArH); 10.26 (s, 1H, CH=O); ^1^H NMR (400 MHz, D_2_O, 298K, ppm): δ = 3.94 (s, 3H; OCH_3_), 7.51 (d, J = 1.9 Hz, 1H; ArH); 7.72 (d, J = 1.9 Hz, 1H; ArH); 10.01 (s, 1H, CH=O).

Bis-(sodium 3-methoxy-5-sulfonate-salicylaldehyde)-thiocarbohydrazone (**bis-TCH**, Na_2_H_4_L^1^∙5.5H_2_O). Sodium 3-methoxy-5-sulfonate-salicylaldehyde (500 mg, 1.97 mmol, 2.0 eq.) was solubilized in a refluxing H_2_O/EtOH mixture (5 mL of H_2_O, 20 mL of EtOH). Thiocarbohydrazide (104 mg, 0.98 mmol, 1.0 eq.) was added to the solution, and after a few minutes, a greenish precipitate formed. The resulting mixture was refluxed for 1 h and 30 min, then cooled to room temperature; the precipitate was filtered off, washed with EtOH, and dried under vacuum. A pale green solid was obtained (538 mg, Y = 80%). ^1^H NMR (400 MHz, d_6_-DMSO, 298K, ppm): δ = 3.83 (s, 6H; OCH_3_), 7.22 (d, J = 1.6 Hz, 2H; ArH), 7.32 (s, br, 1H; ArH), 7.85 (s, br, 1H; ArH), 8.56–8.66 (m, br, ovlp, 2H; CH=N), 9.52 (s, br, 1H; OH), 11.84 (s, br, 1H; OH), 12.04 (s, 2H; NH); ^1^H NMR (400 MHz, D_2_O, 298K, ppm): δ = 3.67 (s, 6H; OCH_3_); 7.09–7.15 (m, br, ovlp, 2H; ArH); 7.33 (s, br, 1H; ArH); 7.63 (s, br, 1H; ArH); 7.82 (s, br, 1H; CH=N); 8.25 (s, br, 1H; CH=N); ^1^H NMR (400 MHz, CD_3_OD, 298K, ppm): δ = 3.94 (s, 6H; OCH_3_); 7.43 (s, 2H; ArH); 7.55 (s, br, 1H; ArH); 8.06 (s, br, 1H; ArH); 8.50–8.64 (m, br, ovlp, 2H; CH=N); ^13^C NMR (101 MHz, d_6_-DMSO, ppm): δ = 55.92, 111.04, 116.07, 117.08, 119.27, 139.31, 141.97, 147.01, 147.50, 149.39, 174.53; IR (cm^−1^): ν = 3429 (m) (OH), 3133 (m) (NH), 1647 and 1612 (m) (C=N). UV/Vis (aqueous 25 mM HEPES buffer at pH 7.4; nm, mol^−1^dm^3^cm^−1^): λ_max_ (ε) = 316 (29000), 241 (35000). MS-ESI (negative ions): m/z (%): 266 (20) [H_4_L]^2−^, 533 (30) [H_5_L]^−^, 555 (100) [NaH_4_L]^−^. Elem. anal. calcd (%) for C_17_H_16_N_4_Na_2_O_10_S_3_∙5.5 H_2_O: C 30.13, H 4.02, N 8.27, S 14.19; found: C 30.05, H 3.79, N 8.17, S 14.46.

Bis-(sodium 3-methoxy-5-sulfonate-salicylaldehyde)-carbohydrazone (**bis-CH**, Na_2_H_4_L^2^∙5H_2_O). Sodium 3-methoxy-5-sulfonate-salicylaldehyde (500 mg, 1.967 mmol, 2.0 eq.) was solubilized in a refluxing H_2_O/EtOH mixture (5 mL of H_2_O, 20 mL of EtOH). Carbohydrazide (88 mg, 0.977 mmol, 1.0 eq.) was added to the solution; work up proceed as for the other ligand (541 mg, Y = 84%). ^1^H NMR (400 MHz, d_6_-DMSO, 298K, ppm): δ = 3.81 (s, 6H; OCH_3_), 7.16 (s, 2H; ArH), 7.50 (s, br, 2H; ArH), 8.40 (s, 2H; CH=N), 10.47 (s, br, 2H, OH), 10.90 (s, br, 2H, NH); ^1^H NMR (400 MHz, D_2_O, 298K, ppm): δ = 3.78 (s, 6H; OCH_3_), 7.20 (d, J = 1.7 Hz, 2H; ArH), 7.47 (s, br, 2H; ArH), 8.06 (s, br, 2H; CH=N); ^1^H NMR (400 MHz, CD_3_OD, 298K, ppm): δ = 3.93 (s, 6H; OCH_3_), 7.41 (d, J = 2 Hz, 2H; ArH), 7.78 (s, br, 2H; ArH), 8.44 (s, br, 2H, CH=N); ^13^C NMR (101 MHz, d_6_-DMSO, ppm): δ = 55.88, 110.37, 117.19, 118.62, 139.38, 142.98, 146.50, 146.90, 151.97. IR (cm^−1^): ν = 3468 (m) (OH), 3200 and 3100 (m) (NH), 1663 (s) (C=O), 1614 and 1586 (m) (C=N). UV/Vis (aqueous 25 mM HEPES buffer at pH 7.4; nm, mol^−1^dm^3^cm^−1^): λ_max_ (ε) = 305 (31400), 236 (42000). MS-ESI (negative ions): m/z (%): 258 (100) [H_4_L]^2−^, 539 (80) [NaH_4_L]^−^. Elem. anal. calcd (%) for C_17_H_16_N_4_Na_2_O_11_S_2_ ∙5 H_2_O: C 31.29, H 4.02, N 8.59, S 9.83; found: C 31.44, H 4.05, N 8.68, S 9.93.

(C1) CuNaH_3_L^1^∙4 H_2_O. Na_2_H_4_L^1^·5.5 H_2_O (80.0 mg, 0.118 mmol, 1.0 eq.) was dissolved in H_2_O/MeOH mixture (2 mL of H_2_O, 10 mL of MeOH; pH ca. 8) at 70 °C. A solution of CuCl_2_ · 2 H_2_O (20.5 mg, 0.120 mmol, 1.0 eq.) in 2 mL of MeOH was added, and the reaction mixture immediately turned dark green (pH ca. 2). After few minutes, a green precipitate formed. The mixture was stirred at room temperature for 2 h. The solvent was partially evaporated, after which the reaction mixture was cooled at t = −20 °C and the solid separated by centrifugation, washed with absolute EtOH, and dried under vacuum. A green powder was obtained (68 mg, Y = 84%). IR (cm^−1^): ν = 3534 and 3410 (w) (OH), 3294 (w) (NH), 1607 and 1586 (m) (C=N); MS-ESI (positive ions): m/z 596 (100) [CuH_5_L]^+^, 618 (60) [Cu_4_H_13_L_3_]^3+^, 640 (50) [Cu_4_Na_3_H_10_L_3_]^3+^. MS-ESI (negative ions): m/z (%): 594 (100) [CuH_3_L]^−^, 616 (15) [CuNaH_2_L]^−^, 657 (15) [Cu_2_HL]^−^. Elem. anal. calcd (%) for C_17_H_15_CuN_4_NaO_10_S_3_·4H_2_O: C 29.59, H 3.36, N 8.12, S 13.94; found: C 29.73, H 3.44, N 8.21, S 13.69.

Crystals of [Cu_2_(NaHL^1^)(H_2_O)_7_]^.^3.5H_2_O were obtained by slow evaporation of the solvent from the mother solution of C1 synthesis, after resting several days at room temperature. Crystallographic data: C_17_H_34_Cu_2_N_4_NaO_20.5_S_3_, triclinic, P-1, a = 9.2804(2) Å, b = 14.1111(3) Å, c = 14.2995(3) Å, 63.355(1), 73.025(1), 77.203(1)°; V = 1591.82(6) Å^3^; Z = 2; d_calc_ = 1.812 mg/cm^3^, F(000) = 890, CuKα radiation (λ = 1.54178), mu = 4.456. Tot. refl. = 25831, hkl range= −11 < h < 11, −17 < k < 17, −17 < l < 17; Theta range 3.524–70.219, unique reflections = 6049, number of parameters = 469, GooF = 1.058, R = 0.037, wR2 = 0.114.

(C2) CuNaH_3_L^2^·5 H_2_O. Na_2_H_4_L^2^·5 H_2_O (100.0 mg, 0.153 mmol, 1.0 eq.) was dissolved in H_2_O/MeOH mixture (3 mL of H_2_O, 7 mL of MeOH; pH ca. 8) at 70 °C. A solution of CuCl_2_·2H_2_O (26.5 mg, 0.155 mmol, 1.0 eq.) in 2 mL of MeOH was added, and the reaction mixture immediately turned dark green (pH ca. 2). After few minutes, a fine green precipitate formed. We then proceeded as with C1. A light green powder was obtained (90 mg, Y = 85%). IR (cm^−1^): ν = 3416 (m) (OH), 3315 (m) (NH), 1651 (s) (C=O), 1604 (m) and 1558 (s) (C=N); MS-ESI (negative ions): m/z (%): 579 (40) [CuH_3_L]^−^, 590 (20) [Cu_2_NaH_5_L_2_]^2−^, 610 (20) [Cu_2_Na_3_H_3_L_2_]^2−^, 641 (20) [Cu_2_HL]^−^. Elem. anal. calcd (%) for C_17_H_15_CuN_4_NaO_11_S_2_·5 H_2_O: C 29.50, H 3.64, N 8.10, S 9.27; found: C 29.68, H 3.36, N 8.00, S 9.28.

(C3) ZnNa_2_H_2_L^1^·6H_2_O·0.33 iPrOH. Na_2_H_4_L^1^ · 5.5 H_2_O (80.3 mg, 0.118 mmol, 1.0 eq.) was dissolved in H_2_O/MeOH mixture (2 mL of H_2_O, 10 mL of MeOH; pH ca. 8) at 70 °C. A solution of Zn(CH_3_COO)_2_·2H_2_O (26 mg, 0.118 mmol, 1.0 eq.) in 3 mL of MeOH was added, and the reaction mixture (pH ca. 7) was stirred at room temperature for 2 h. The solvent was partially evaporated, and 2-propanol (15 mL) was added; immediately, a beige precipitate formed. The suspension was cooled to −20 °C, and the precipitate was filtered off, washed with 2-propanol and Et_2_O, and dried under vacuum. A beige powder was obtained (69 mg, Y = 76%). IR (cm^−1^): ν = 3420 (m) (OH), 3160 (m) (NH), 1603 (m) (C=N). MS-ESI (negative ions): m/z (%): 330 (30) [Zn_2_L]^2−^, 597 (50) [ZnH_3_L]^−^, 608 (20) [Zn_2_NaH_5_L_2_]^2−^, 619 (20) [ZnNaH_2_L]^−^, 630 (20) [Zn_2_Na_3_H_3_L_2_]^2−^, 641 (20) [ZnNa_2_HL]^−^, 660 (15) [Zn_2_HL]^−^. Elem. anal. calcd (%) for C_17_H_14_N_4_Na_2_O_10_S_3_Zn·0.33 (CH_3_)_2_CHOH∙6 H_2_O: C 28.08, H 3.75, N 7.28, S 12.49; found: C 27.87, H 3.21, N 7.03, S 12.48.

(C4) ZnNa_2_H_2_L^2^∙5 H_2_O∙0.5iPrOH. Na_2_H_4_L^2^∙5 H_2_O (99.7 mg, 0.153 mmol, 1.0 eq.) was dissolved in H_2_O/MeOH mixture (4 mL of H_2_O, 7 mL of MeOH; pH ca. 8) at 70 °C. A solution of Zn(CH_3_COO)_2_ · 2 H_2_O (33.3 mg, 0.152 mmol, 1.0 eq.) in 3 mL of MeOH was added, and the reaction mixture (pH ca. 7) was stirred at room temperature for 4 h. We then proceeded as with C3. An off-white powder was obtained (91 mg, Y = 80%). IR (cm^−1^): ν = 3418 (m) (OH), 3200 (m) (NH), 1600 (m) (C=O), 1555 (m) (C=N); MS-ESI (negative ions): m/z (%): 289 (50) [ZnH_2_L]^2−^, 581 (20) [ZnH_3_L]^−^, 592 (15) [Zn_2_NaH_5_L_2_]^2−^, 603 (15) [ZnNaH_2_L]^−^, 614 (15) [Zn_2_Na_3_H_3_L_2_]^2−^, 625 (15) [ZnNaH_2_L]^−^. Elem. anal. calcd (%) for C_17_H_14_N_4_Na_2_O_11_S_2_Zn · 0.5 (CH_3_)_2_CHOH · 5 H_2_O: C 29.79, H 3.78, N 7.51, S 8.60; found: C 29.68, H 3.82, N 7.46, S 8.60.

(C5) Cu_2_NaHL^1^∙7H_2_O. Na_2_H_4_L^1^∙5.5 H_2_O (188.0 mg, 0.277 mmol, 1.0 eq.) was dissolved in 13 mL of MeOH (pH ca. 8) at 30 °C. A solution of CuCl_2_∙2 H_2_O (95 mg, 0.557 mmol, 2.0 eq.) in 3 mL of MeOH was added, immediately forming a dark green suspension (pH ca. 2–3) formed, which was stirred at room temperature for 2 h. The resulting suspension was cooled to −20 °C, and the solid was separated by centrifugation, washed with MeOH, and dried under vacuum. A dark green hygroscopic powder was obtained (74 mg, Y = 33%). IR (cm^−1^): ν = 3379 (m) (OH), 3160 (m) (NH), 1598 (s) and 1546 (m) (C=N); MS-ESI (negative ions): m/z (%): 328 (80) [Cu_2_L]^2−^, 656 (80) [Cu_2_HL]^−^. Elem. anal. calcd (%) for C_17_H_13_Cu_2_N_4_NaO_10_S_3_∙7 H_2_O: C 25.34, H 3.38, N 6.95, S 11.94; found: C 25.33, H 2.98, N 6.94, S 11.62.

(C6) Cu_2_NaHL^2^∙7H_2_O. Na_2_H_4_L^2^∙5 H_2_O (150.0 mg, 0.230 mmol, 1.0 eq.) was dissolved in H_2_O/MeOH mixture (4 mL of H_2_O, 10 mL of MeOH; pH ca. 8) at 70 °C. A solution of CuCl_2_∙2 H_2_O (79.0 mg, 0.463 mmol, 2.0 eq.) in 3 mL of MeOH was added, and a dark green suspension (pH ca. 2) formed immediately; we then proceeded as with C5. A green hygroscopic powder was obtained (158 mg, Y = 87%). IR (cm^−1^): ν = 3400 (m) (OH), 3211 (m) (NH), 1596 (s) (C=O), 1547 (m) (C=N); MS-ESI (negative ions): m/z (%): 640 (100) [Cu_2_HL]^−^. Elem. anal. calcd (%) for C_17_H_13_Cu_2_N_4_NaO_11_S_2_ · 7 H_2_O: C 25.86, H 3.45, N 7.10, S 8.12; found: C 26.13, H 3.22, N 7.09, S 8.05.

(C7) Zn_2_(Na_2_HL^1^)(OAc)∙7H_2_O. Na_2_H_4_L^1^·5.5 H_2_O (151.0 mg, 0.223 mmol, 1.0 eq.) was dissolved in 11 mL of MeOH (pH ca. 8) at 30 °C. A solution of Zn(CH_3_COO)_2_ ·2 H_2_O (98.0 mg, 0.446 mmol, 2.0 eq.) in 3 mL of MeOH was added, and the reaction mixture (pH ca. 6) was stirred at room temperature for 2 h. The solvent was partially evaporated, and 2-propanol (15 mL) was added: a fine precipitate formed, which was filtered off, washed with cold 2-propanol, and dried under vacuum. A beige hygroscopic powder was obtained (91 mg, Y = 46%). ^1^H NMR (400 MHz, D_2_O, 298K, ppm): δ = 1.92 (s, 3H; CH_3_, OAc), 3.70–3.85 (m, br, 6H; OCH_3_), 7.15–7.35 (m, br, 4H; ArH), 7.96 (s, br, 1H; CH=N), 8.68 (s, br, 1H; CH=N); IR (cm^−1^): ν = 3390 (m) (OH), 3200 (m) (NH), 1600 and 1549 (m) (C=N); MS-ESI (negative ions): m/z (%): 330 (100) [Zn_2_L]^2−^, 440 (20) [Zn_4_HL_2_]^3−^, 660 (30) [Zn_2_HL]^−^, 682 (30) [Zn_2_NaL]^−^. Elem. anal. calcd (%) for C_19_H_16_N_4_Na_2_O_12_S_3_Zn_2_ · 7 H_2_O: C 25.60, H 3.39, N 6.28, S 10.79; found: C 25.57, H 3.07, N 5.84, S 11.37.

(C8) Zn_2_(Na_2_HL^2^)(OAc)·5H_2_O. Na_2_H_4_L^2^·5 H_2_O (135.0 mg, 0.207 mmol, 1.0 eq.) was dissolved in H_2_O/MeOH mixture (4 mL of H_2_O, 10 mL of MeOH; pH ca. 8) at 70 °C. A solution of Zn(CH_3_COO)_2_ · 2 H_2_O (91.0 mg, 0.414 mmol, 2.0 eq.) in 3 mL of MeOH was added and the reaction mixture (pH ca. 7) was stirred at room temperature for 4 h; then, we proceeded as with C7. A brown hygroscopic powder was obtained (69 mg, Y = 39%). IR (cm^−1^): ν = 3408 (m) (OH), 3221 (m) (NH), 1595 (s) (C=O), 1558 (m) (C=N); MS-ESI (negative ions): m/z (%): 290 (50) [ZnH_2_L]^2−^, 322 (100) [Zn_2_L]^2−^, 429 (45) [Zn_4_HL_2_]^3−^, 644 (40) [Zn_2_HL]^−^, 653 (30) [Zn_4_NaHL_2_]^2−^. Elem. anal. calcd (%) for C_19_H_16_N_4_Na_2_O_13_S_2_Zn_2_ · 5H_2_O: C 26.68, H 3.06, N 6.55, S 7.50; found: C 27.03, H 3.24, N 6.80, S 7.90.

### 3.2. Studies in Solution

The UV–visible spectra were collected using a Thermo Scientific Evolution 260 Bio spectrophotometer equipped with a Peltier temperature controller. Aqueous buffer stock solutions (50 mM) were prepared as follows: acetate buffer solutions (pH = 3.98, 4.62, and 5.00) were prepared by mixing appropriate volumes of AcOH 50 mM and AcONa·3H_2_O 50 mM solutions. MES (pH = 5.51, 6.00, and 6.49), HEPES (pH = 7.00, 7.48, and 8.01) and CHES (pH = 8.62, 9.01, 9.51, and 10.00) buffer solutions were obtained by suspending appropriate amounts of solid MES/HEPES/CHES in water, adding aqueous NaOH 100 mM until the desired pH was reached. The pH was measured using a Crison Basic 20 pH meter connected with a Hamilton glass electrode. Calibration of the glass electrode was carried out daily. All solutions were prepared using double-distilled water. For all the solutions, the ionic strength was 0.1 M NaCl.

Stock solutions of **bis-TCH** and **bis-CH** were prepared in water at a concentration of ca. 1 mM. Stock solutions of CuCl_2_·2H_2_O and ZnCl_2_ (ca. 10 mM) were prepared in water by weight of the salts and their titre determined using standard EDTA solutions [20]. HCl and NaOH stock solutions were prepared in water at a concentration of 100 mM. All titrant solutions were prepared by dilution of the stock solutions in double-distilled water.

### 3.3. UV–Visible Spectrophotometric Titrations

Samples used for the study of protonation equilibria of the ligands were prepared as follows. Solutions of the ligands (C_L_ = 17 μM; volume 2.6 mL) at different pH were prepared in the cuvette by diluting the ligand stock solutions using buffer solutions at the desired pH. For each solution, the absorption spectrum was collected in the 220–600 nm range at 298.2 K. The spectral data collection was carried out immediately after the preparation of the samples and repeated after 3 and 10 min for all the samples at different pH values to check the stability of the ligand. For all systems the collected spectra did not change within 10 min after ligand addition. The complex formation equilibria at pH 2.5 or 7.4 of **bis-TCH** and **bis-CH** with Cu(II) and Zn(II) ions were studied by direct spectrophotometric titrations of solutions of the ligands with the metal ions, and the samples were prepared as follows. Appropriate aliquots of the stock solutions of the ligands were diluted in a quartz cuvette of 1 cm path length using HEPES buffer (pH 7.4) or by adding HCl aqueous solution (pH 2.5) to obtain ca. 17 µM ligand solutions in a total volume of 2.6 mL. The obtained ligand solutions were titrated with Cu(II) or Zn(II) titrant solutions (ca. 442 µM) up to a metal/ligand ratio of 3 for the studies at pH 7.4 and up to a Cu(II):ligand ratio of 5 (for Na_2_H_4_L^1^ · 5.5 H_2_O) and 7 (for Na_2_H_4_L^2^ · 5 H_2_O) for the studies at pH 2.5. For each solution, the absorption spectrum was collected in the 220–600 nm range at 298.2 K. All experiments were performed in triplicate.

The logarithms of the apparent stability constants were calculated from the spectral dataset using HypSpec2014 software [21]. For each system, data from different titrations were treated together. In all titrations, the molar spectrum of the ligand at the appropriate pH has been used as the fixed parameter. All equilibria were studied at fixed pH, and therefore the determined log *β* values are logarithms of conditional formation constants. In the speciation models the L component identifies the studied ligand irrespective of its protonation state; for this reason, the charges of the complexes are omitted. Speciation diagrams were calculated using the Hyss2009 software [22]. Where appropriate, the complexation equilibria of Cu(II) with HEPES were considered in the calculations, using a conditional log *β* for the Cu(II)/HEPES adduct at pH 7.4 of 2.91 (calculated from [23]). No significant competing role of HEPES in zinc complexation is documented in the literature [24].

### 3.4. Potentiometric Titrations

Glass-electrode potentiometric titrations of **bis-TCH** and **bis-CH** were carried out in aqueous solution at *T* = 298.2 ± 0.1 K and *I* = 0.1 M (KCl) under a stream of N_2_, using 1.5 mL samples. The potentiometric titrations were carried out using an automatic Metrohm OMNIS titrator provided with a Metrohm semi-micro glass electrode. The system was controlled by a PC to monitor the attainment of the equilibrium through pH measurements vs. time and the addition of titrant aliquots. The Metrohm combined glass electrode was calibrated in terms of [H^+^] (hereafter, pH = −log[H^+^]) by titrating HCl solutions with KOH solutions previously standardized against potassium hydrogen phthalate [25,26]. The p*K_w_* values were found to be in the 13.75–13.77 range. The protonation constants of Na_2_H_4_L^1^·5.5 H_2_O and Na_2_H_4_L^2^·5 H_2_O were determined by alkalimetric titration of 3 samples (3.0–4.5 mM). All samples were prepared by weight of the pure solids and dissolved in freshly prepared, N_2_-fluxed, double-distilled water. Potentiometric data treatment was carried out using Hyperquad2013 software [21]. Distribution diagrams were obtained using Hyss2009 software [22].

### 3.5. Cell Line and Culture Conditions

U937 cells (ATCC, CRL-1593.2) were obtained from the American Tissue Culture Collection (Rockville, MD, USA) and were cultured in Roswell Park Memorial Institute Medium (RPMI-1640) (Euroclone, Italy); Hs27 cells (ATCC, CRL1634) were grown in DMEM. Both media were supplemented with 10% (*v*/*v*) foetal bovine serum (Euroclone, Italy), 1% L-glutamine (2 mM) (Euroclone, Italy), and 1% penicillin (100 U/mL)/streptomycin (100 µg/mL) (Euroclone, Italy), added with 10% (*v*/*v*) foetal bovine serum (Euroclone, Italy), 1% L-glutamine (2 mM) (Euroclone, Italy), and 1% penicillin (100 U/mL)/streptomycin (100 µg/mL) (Euroclone, Italy). Flasks were maintained at 37 °C and 5% CO_2_ in a humidified atmosphere. Cultures media were refreshed every two or three days during sub-culturing.

The antiproliferative activity of the ligands and the complexes **C1**–**C4** was evaluated in vitro on U937 and Hs27 cell lines. In the exponential growth phase, cells were seeded at 5 × 10^4^/mL into 12-well flat-bottom microplates. Cells were cultured in complete medium supplemented with 10% FBS, 1% L-glutamine, and 1% penicillin/streptomycin at 37 °C, 5% CO_2_. After seeding, different concentrations of compounds in the range from 0 to 100 µg/mL in a sterile 1X PBS solution were added to the wells and incubated for 24, 48, and 72 h. A negative control was obtained by treating cells with 1X PBS. To detect whether the treatments caused a defect in cell viability, following 24, 48, and 72 h treatments with the compounds, cells were stained with trypan blue (TB, 0.4%) (Gibco™, Thermofisher Scientific, Waltham, MA, USA). Two independent experiments were performed, and a Bürker chamber was used for cell counts. The GI_50_ value, i.e., the concentration causing a 50% reduction in the cell number in comparison with control cells, was calculated taking into account the guidelines of the US National Cancer Institute (NCI) [27]. Values are reported as the mean of the GI_50_ calculated on two independent experiments.

### 3.6. Crystallography

Single crystals were mounted on a glass fibre, and the intensity data were collected with a SMART APEX2 diffractometer equipped with a Bruker AXS CCD detector. The SAINT1986 [28] software was used for the integration of reflection intensities and scaling, and SADABS1996 [29] was used for the absorption correction. The structures were solved by direct methods using SIR97 [30] and refined by full-matrix least squares on all F2 using SHELXL97 [31] implemented in the WinGX package [32]. All the non-hydrogen atoms in the molecules were refined anisotropically. The hydrogen atoms were partly found and partly placed in ideal positions using riding models. The structures were solved by direct methods and difference Fourier synthesis using the SHELX suite of programs as implemented within WINGX1999 software. Thermal ellipsoid plots were generated using the program ORTEP-333 integrated within the WinGX suite of programs.

The crystallographic data were as follows: C17, H34, Cu2, N4, Na, O20.5, S3; triclinic, P-1, a = 9.2804(2) Å, b = 14.1111(3) Å, c = 14.2995(3) Å, 63.355(1), 73.025(1), 77.203(1)°. V = 1591.82(6) Å^3^; Z = 2; d_calc_ = 1.812 mg/cm^3^, F(000) = 890, CuKα radiation (λ = 1.54178), mu = 4.456. Tot. refl. = 25831, hkl range = −11 < h < 11, −17 < k < 17, −17 < l < 17. Theta range 3.524–70.219, unique reflections = 6049, number of parameters = 469, GooF = 1.058, R = 0.037, wR2 = 0.114.

## 4. Conclusions

To study cytotoxic activity, it is crucial to obtain compounds that are soluble in water and stable in solution. The introduction of the sulfonic group makes the ligands **bis-TCH** and **bis-CH** very soluble in water. Interestingly, the study of their speciation and coordinating behaviour towards biologically relevant Cu(II) and Zn(II) ions highlighted that **bis-TCH** and **bis-CH**, but in particular **bis-TCH**, are already able to complex with Cu(II) ions at unusually low pH values, giving rise to mono- and polymetallic complexes. The possibility of obtaining bimetallic complexes was unequivocally demonstrated by the X-ray diffraction analysis of [Cu_2_(NaHL^1^)(H_2_O)_7_]^.^3.5H_2_O. This X-ray structure also suggests the interesting possibility that cationic species, such as sodium in this example, can bridge complex units to form coordination polymers; coordination polymers appear to promise new interesting applications in various fields of research. The monometallic and bimetallic complexes of copper(II) and zinc(II) with **bis-TCH** and **bis-CH** were isolated, and they have good solubility in water. The introduction of the sulfonic group in the molecular skeleton could limit the cytotoxicity of the new compounds, for example, by limiting their ability to cross the cell membrane [33], but it is reasonable to suppose that the formation of the metal complex, by increasing lipophilicity, could mitigate these possible negative effects. Indeed, the preliminary cytotoxicity measurements with **C1**–**C4** go in this direction: the complexes, and in particular the copper(II) ones, show a degree of cytotoxicity that is decidedly higher than that of the corresponding free ligands **bis-TCH** and **bis-CH**. However, these values are modest compared to the nanomolar activity presented by some copper(II) thiosemicarbazone complexes [34]. It is also interesting to note that the more cytotoxic complex **C2** is obtained from the bis-carbohydrazone **bis-CH** and not from the bis-thiocarbohydrazone **bis-TCH** [28]. It is a widely shared opinion that once the Cu(II) ion is transported inside the cell as a complex, it is reduced to Cu(I) and then becomes active. Considering the remarkably high affinity of **bis-TCH** for Cu(II), which we have identified by means of the solution studies, it is possible that in the case of **C1**, the reduction of the metal ions by the endogenous reductants is difficult, leading to limited activity compared to **C2**; however, at present, this remains merely an intriguing working hypothesis.

In conclusion, we have synthesized and studied the coordinating behaviour of two ligands that, due to their coordinative potential in aqueous solution, may be interesting for synthesizing unusual metal-organic structures and have possible applications in bioinorganic chemistry.

## Figures and Tables

**Table 1:** Overview of the complexes **C1**–**C8**.

Metal	Ligand	M:L Ratio	Complex	M:L Ratio	Complex
Cu(II)	bis-TCH	1:1	C1	2:1	C5
	bis-CH	1:1	C2	2:1	C6
Zn(II)	bis-TCH	1:1	C3	2:1	C7
	bis-CH	1:1	C4	2:1	C8

**Table 2:** Logarithms of the proton dissociation constants (p*K_a_*) of the ligands determined in aqueous solution at *T* = 298.2 K by UV–visible spectrophotometry and pH potentiometry. Standard deviations are reported in parentheses.

	bis-TCH		bis-CH	
	UV–Visible Spectrophotometry	pH Potentiometry	UV–Visible Spectrophotometry	pH Potentiometry
p*Ka2*	-	-	-	1.70(10)
p*Ka3*	6.78(1)	7.08(4)	7.34(1)	7.77(3)
p*Ka4*	8.77(1)	8.54(4)	8.58(1)	8.45(3)
p*Ka5*	-	11.29(2)	-	-

**Table 3:** Logarithms of the conditional formation constants of the Cu(II) complexes with the ligands in aqueous solution at pH = 2.5 and *T*= 298.2 K. Standard deviations are reported in parentheses.

	bis-TCH	bis-CH
Log *β*([Cu_3_L_4_])	38.6(1)	-
Log *β*([CuL])	-	6.26(2)
Log *β*([Cu_3_L_2_])	26.4(1)	-
Log *β*([Cu_2_L])	-	11.30(2)

**Table 4:** Logarithms of the conditional formation constants of the Zn(II) complexes with the ligands in aqueous 25 mM HEPES buffer at pH 7.4 and at 25 °C. Standard deviations are reported in parentheses.

	bis-TCH	bis-CH
Log *β*([ZnL])	8.40(6)	7.08(3)
Log *β*([Zn_2_L])	15.29(10)	13.61(4)

**Table 5:** GI_50_ values (µg/mL) obtained in the U937 and Hs27 cell lines after 24, 48, and 72 h treatment with the two ligands and the complexes **C1**–**C4**.

Compound	GI_50_ Value (µg/mL)					
	Hs27			U937		
	24 h	48 h	72 h	24 h	48 h	72 h
**bis-TCH**	-	-	-	-	-	98.00 ± 2.82
**bis-CH**	-	-	-	-	-	46.50 ± 4.94
**C1**	-	-	69.50 ± 3.18	78.50 ± 6.36	82.75 ± 0.35	76.50 ± 2.12
**C2**	74.25 ± 11.66	38.00 ± 4.94	36.50 ± 0.71	89.75 ± 0.35	53.50 ± 4.24	34.25 ± 10.25
**C3**	-	-	91.00 ± 7.77	-	-	-
**C4**	-	-	-	-	-	-

## Data Availability

Data are contained within the article.

## Supplementary Materials

Supporting information can be downloaded at: https://www.mdpi.com/article/10.3390/ijms251910831/s1.
