# Supplementary material for: Synthesis and Preliminary Studies for In Vitro Biological Activity of Two New Water-Soluble Bis(thio)carbohydrazones and Their Copper(II) and Zinc(II) Complexes
Source: Int J Mol Sci. 2024 Oct 9;25(19):10831. doi: 10.3390/ijms251910831 (PMC11476552; doi:10.3390/ijms251910831)
Supplement: Supplementary file 1 [file ijms-25-10831-s001.zip › ijms-3229617-supplementary.docx]

**SUPPORTING INFORMATION**

**Synthesis and preliminary studies for *in vitro* biological activity of two new water soluble bis(thio)carbohydrazones and their copper(II) and zinc(II) complexes**

Alessio Zavaroni,^[a]^ Elena Riva,^[a]^ Valentina Borghesani,^[a]^ Greta Donati,^[b]^ Federica Santoro,^[b]^ Vincenzo Maria D’Amore,^[b]^ Matteo Tegoni,^[a]^ Giorgio Pelosi,^[a,c]^ Annamaria Buschini,^[a,c]^ Dominga Rogolino,^[a]^ and Mauro Carcelli*^[a]^

[a] Department of Chemistry, Life Sciences and Environmental Sustainability, University of Parma, Parco Area delle Scienze 17/A, Parma, Italy
E-mail: mauro.carcelli@unipr.it

[b] Department of Pharmacy, University of Naples, Via Domenico Montesano, 49, 80131, Napoli, Italy

[c] Centre for Molecular and Translational Oncology, University of Parma, Parco Area delle Scienze 11/A, Parma, Italy

Supporting information for this article is given via a link at the end of the document.

**Figure S1 IR bis-TCH**

**
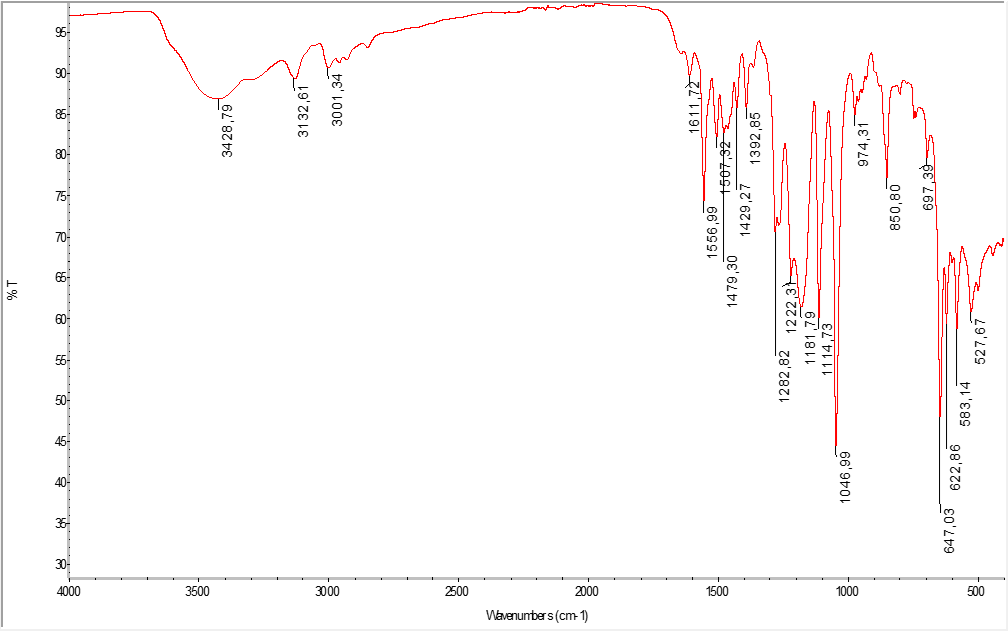
**

**Figure S2 IR bis-CH**

**
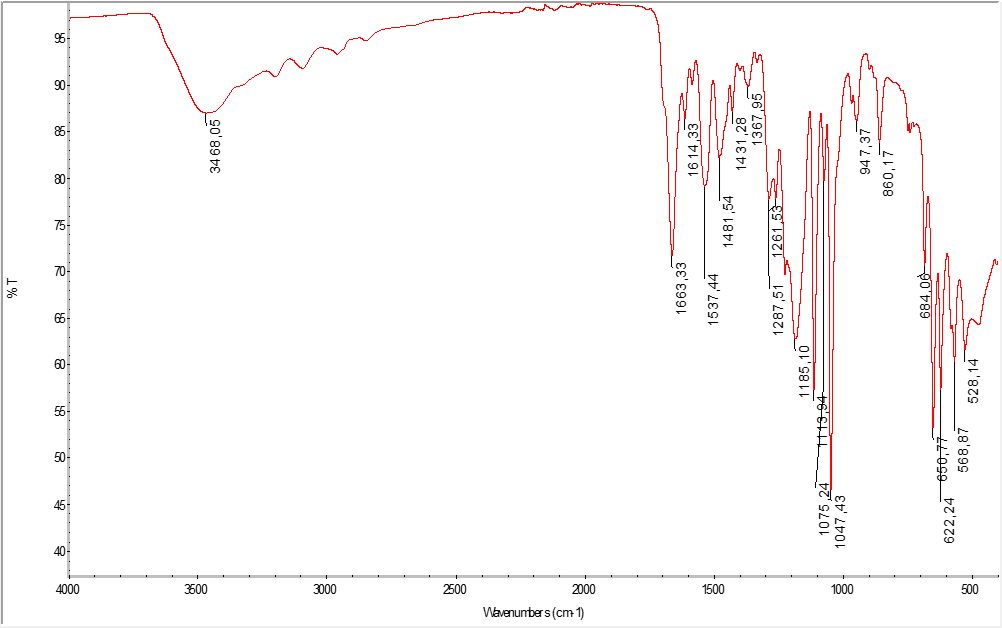
**

**Figure S3. A)** ^1^H NMR and **B)** ^13^C NMR spectrum for **Na_2_H_4_L^1^** (**bis-TCH**) registered in d_6_-DMSO.

**A)**

**B)**

**Figure S4.** ^1^ **A)** ^1^H NMR and **B)** ^13^C NMR spectrum for **Na_2_H_4_L^2^** (**bis-CH**) registered in d_6_-DMSO.

**A)**

**B)**

**Figure S5.** TGA plot (temperature range: 30–300 °C, heating rate: 10 °C/min). A) Na_2_H_4_L^1^·5.5H_2_O (**bis-TCH**): the first event at 30-50 °C (weight loss: 2.769 %) is consistent with the loss of one water molecule; the second event at 50-80 °C (weight loss: 2.619 %) is related to the loss of another water molecule. The third event at 80-170 °C (weight loss: 9.069 %) is consistent with the loss of 3.5 water molecules. B) Na_2_H_4_L^2^·5H_2_O (**bis-CH**): the first event at 30-80 °C (weight loss %: 6.778 %): loss of 2.5 water molecules; second event at 80-300 °C (weight loss: 7.890 %): loss of 2.5 water molecules.

**A)**


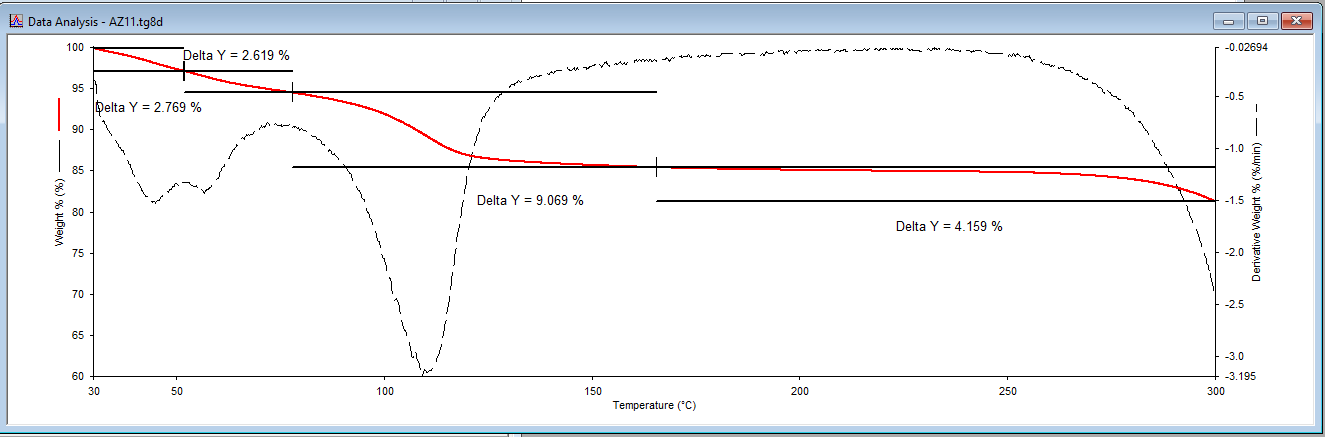


**B)**


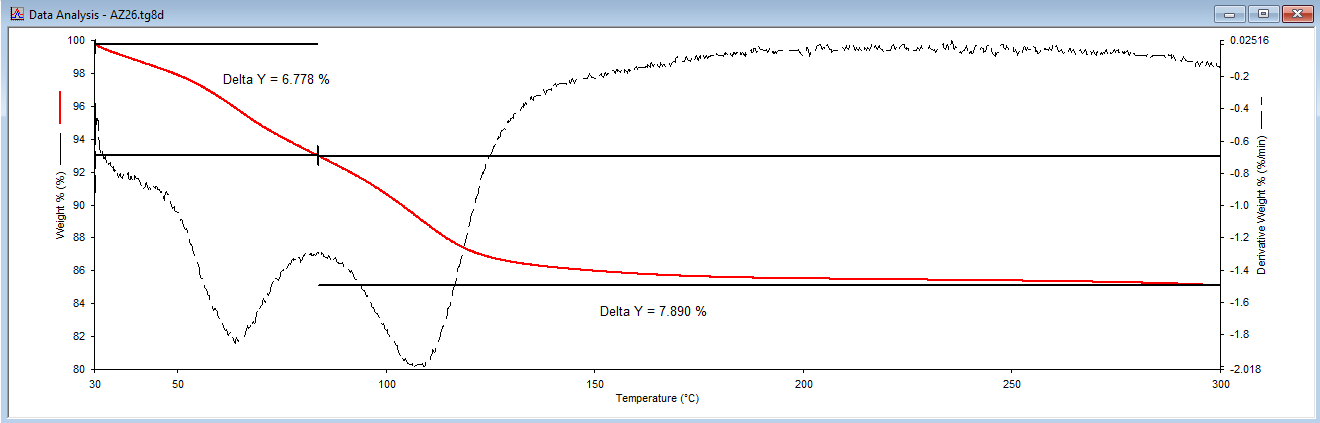


**Figure S6.** ^1^H NMR spectrum of **C7** in D_2_O at 298 K.

**Figure S7.** ESI-MS spectrum (positive ions) for complex **C1** in methanol. Bottom: calculated isotopic distributions.


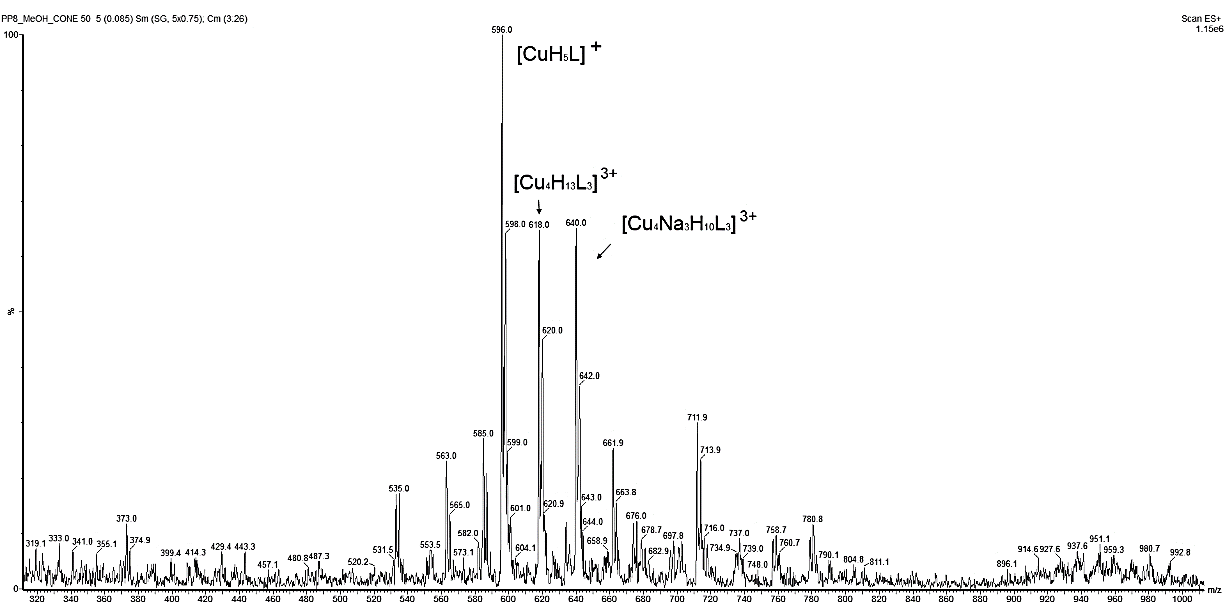

**Figure S8.** Top: ESI-MS spectrum (negative ions) for complex **C2** in methanol. Bottom: calculated isotopic distributions.


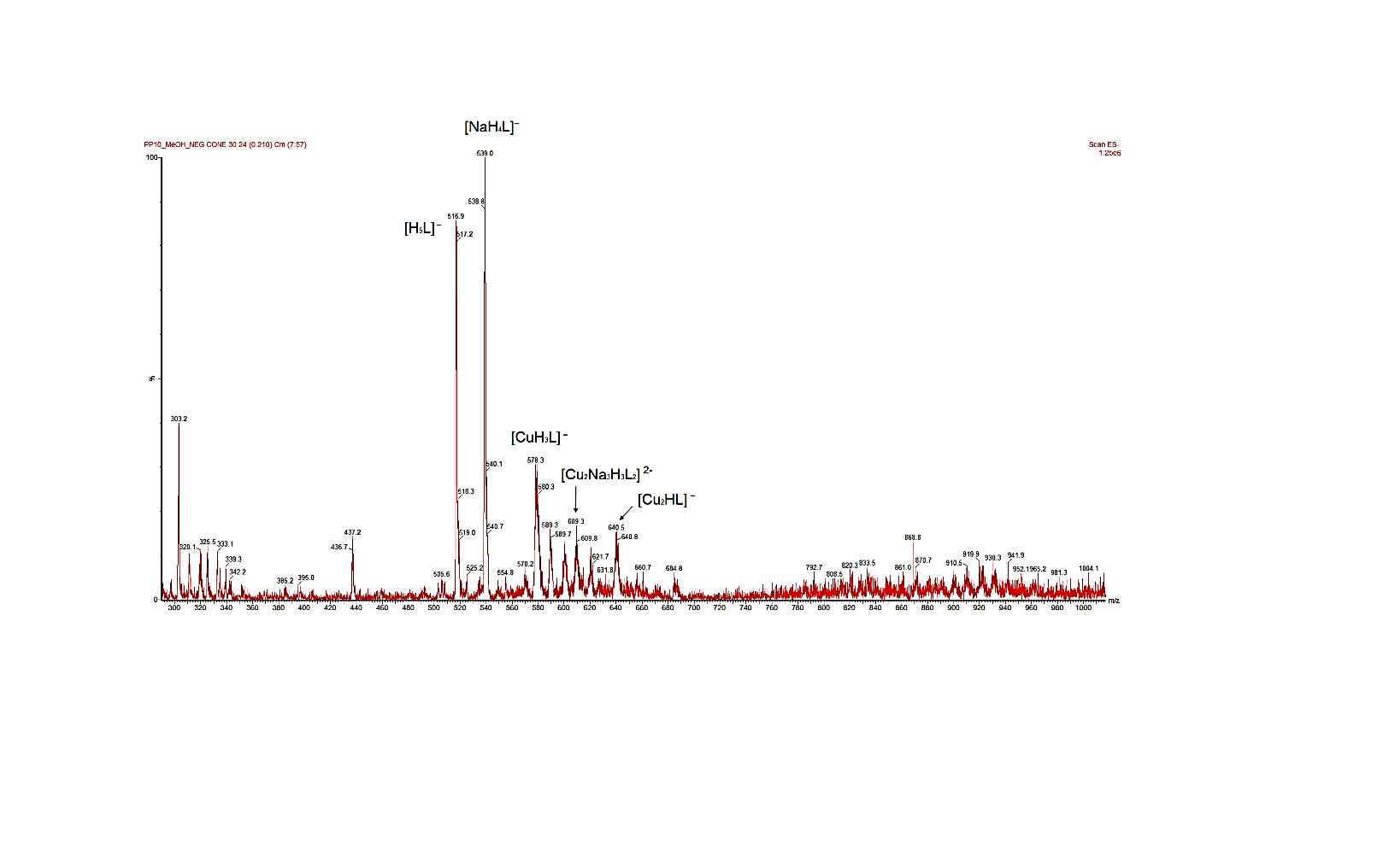

**Figure S9.** ESI-MS spectrum (negative ions) for complex **C3** in methanol. Bottom: calculated isotopic distributions.


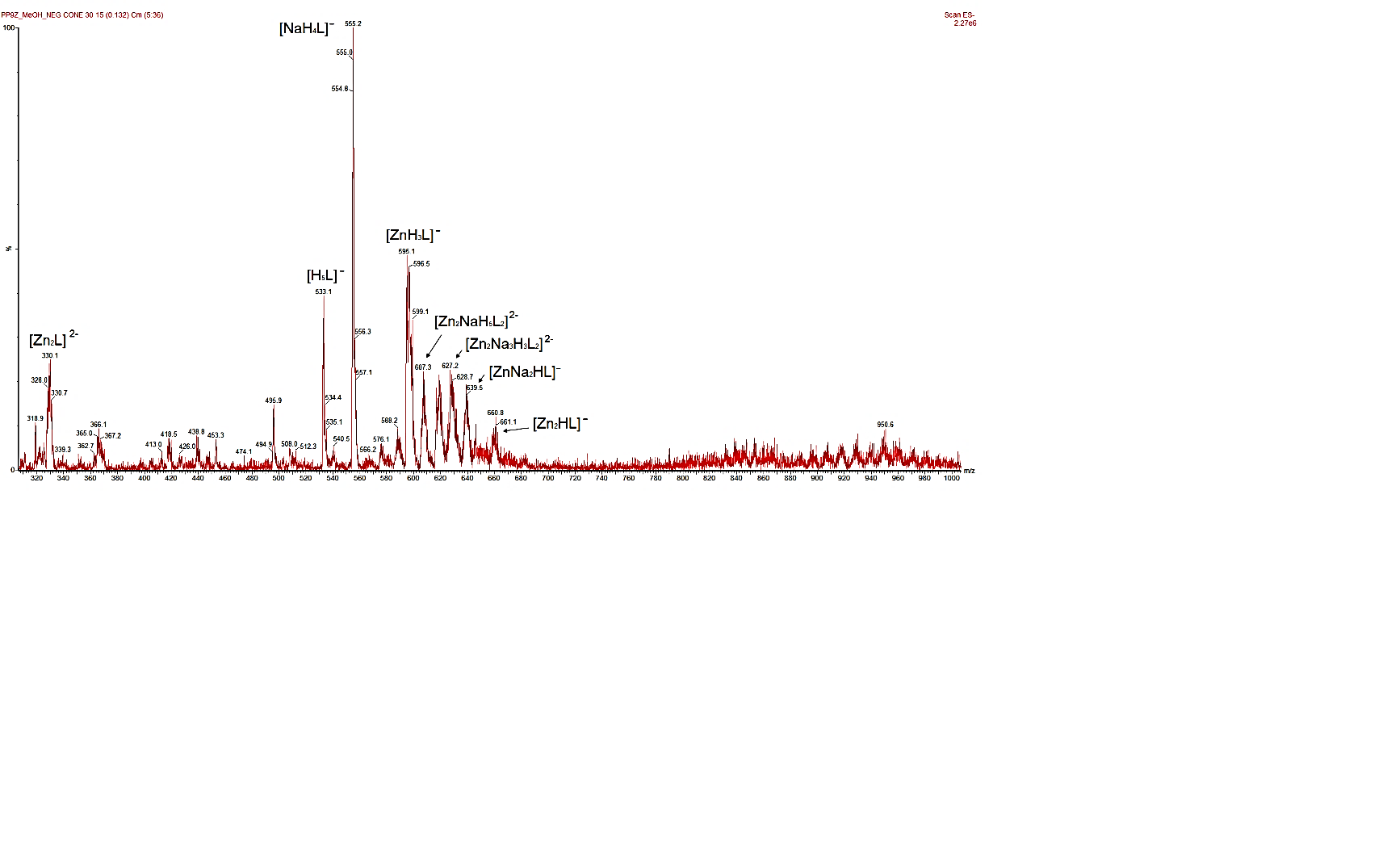

**Figure S10.** ESI-MS spectra (negative ions) for complex **C4** in methanol. Bottom: calculated isotopic distributions.


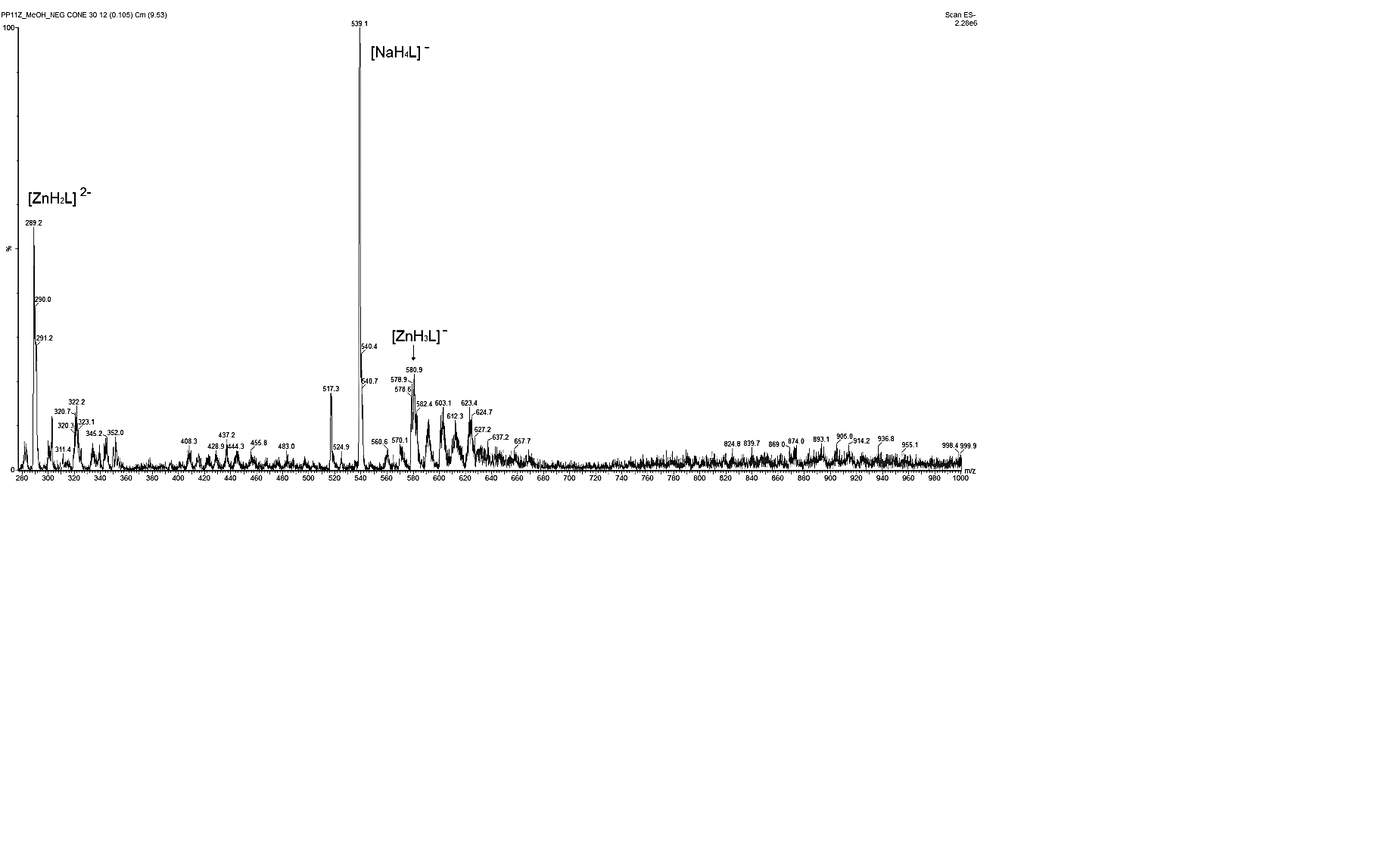

**Figure S11.** ESI-MS spectra (negative ions) for complex **C5** in methanol. Bottom: calculated isotopic distributions.


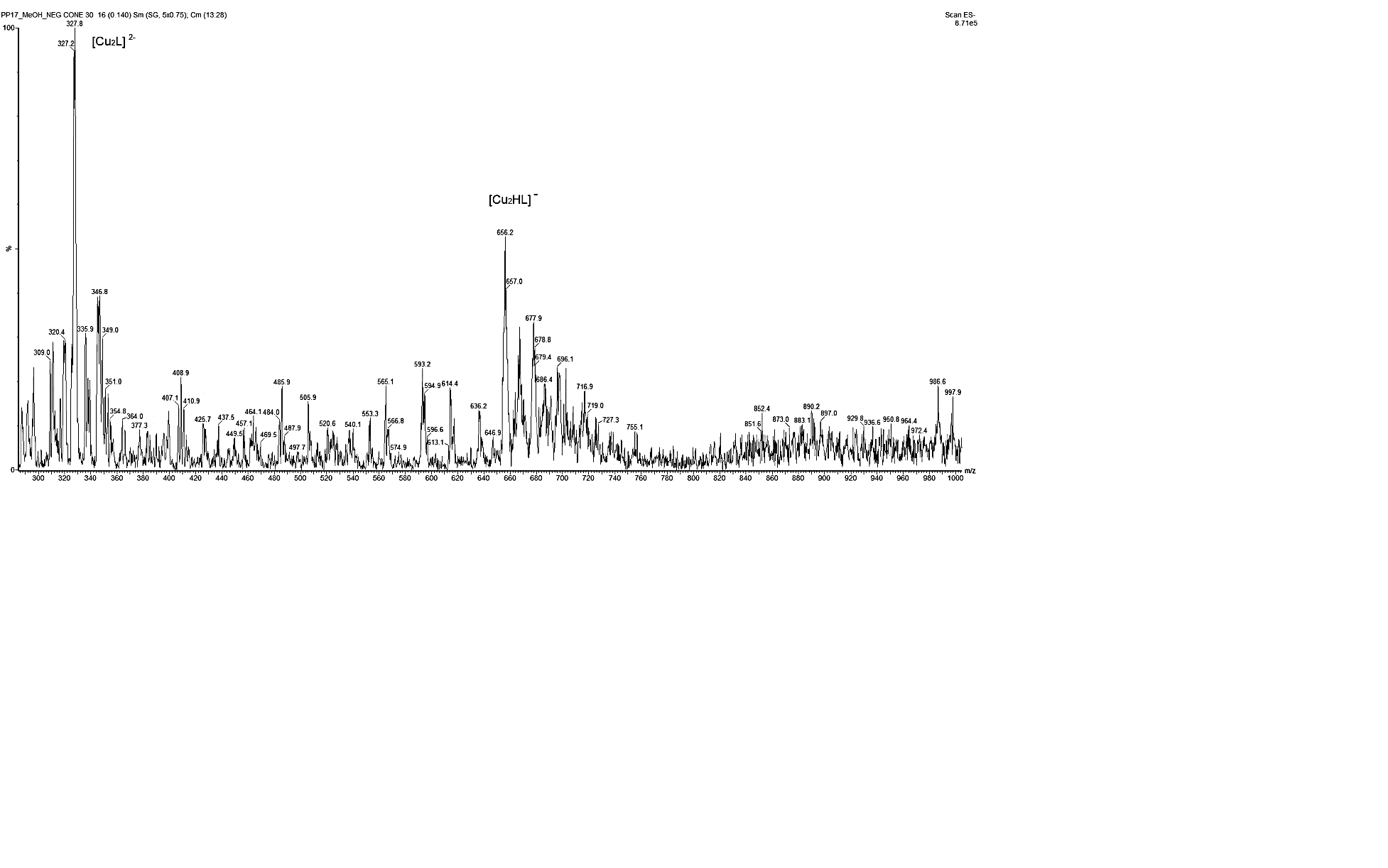

**Figure S12.** ESI-MS spectrum (negative ions) for complex **C6** in methanol. Bottom: calculated isotopic distributions.


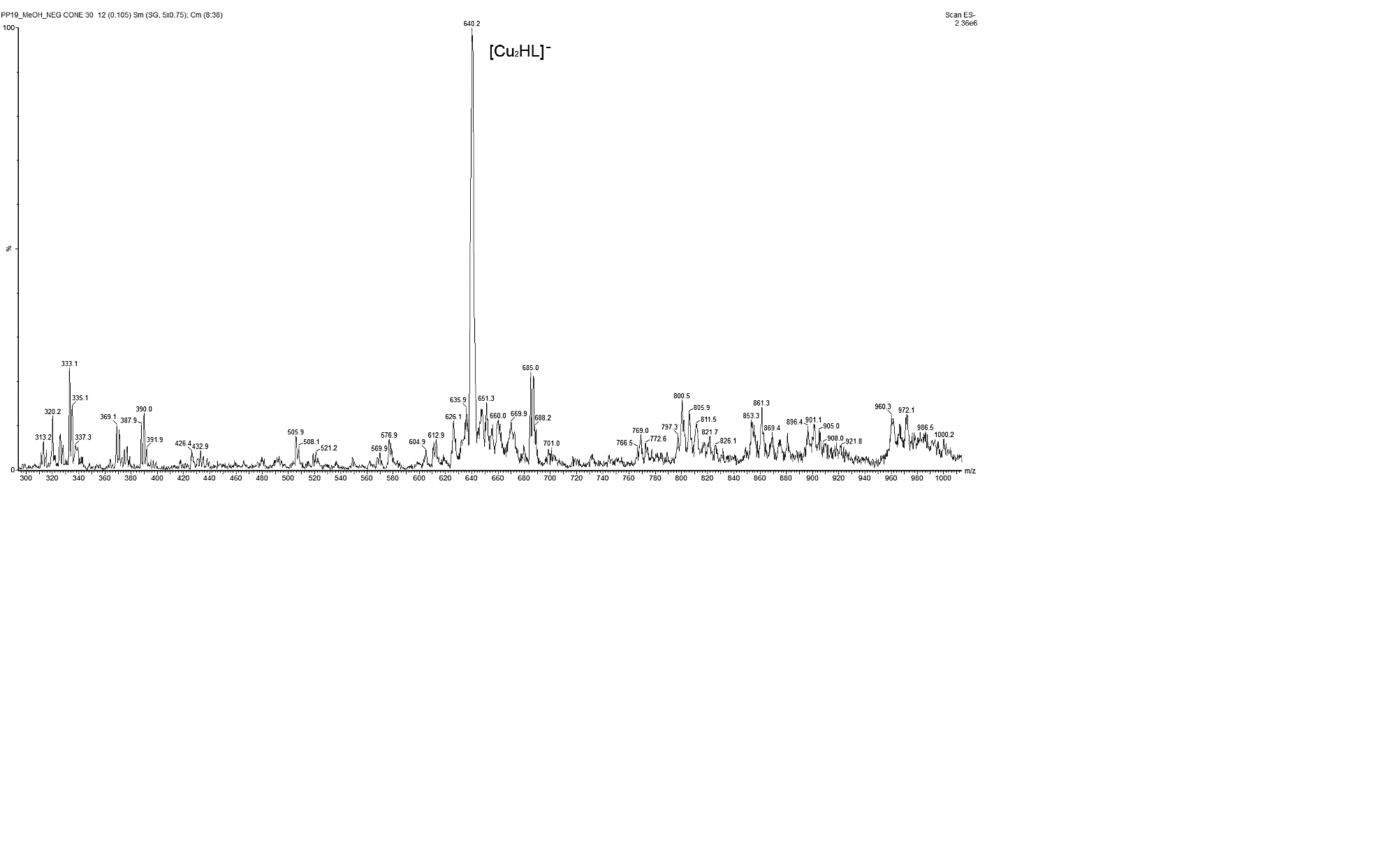

**Figure S13.** ESI-MS spectra (negative ions) for complex **C7** in methanol. Bottom: calculated isotopic distributions.


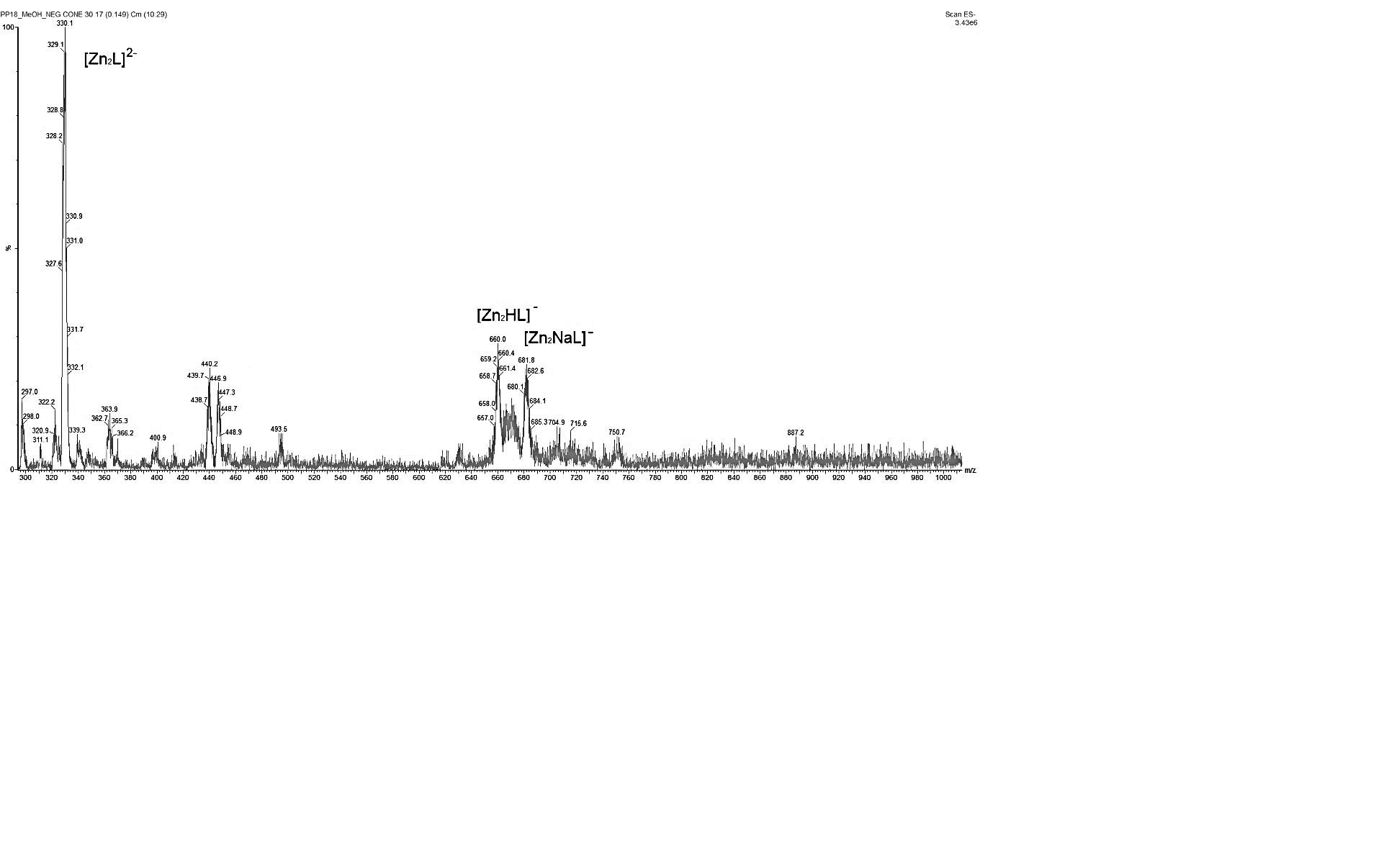

**Figure S14.** ESI-MS spectrum (negative ions) for complex **C8** in methanol. Bottom: calculated isotopic distributions.


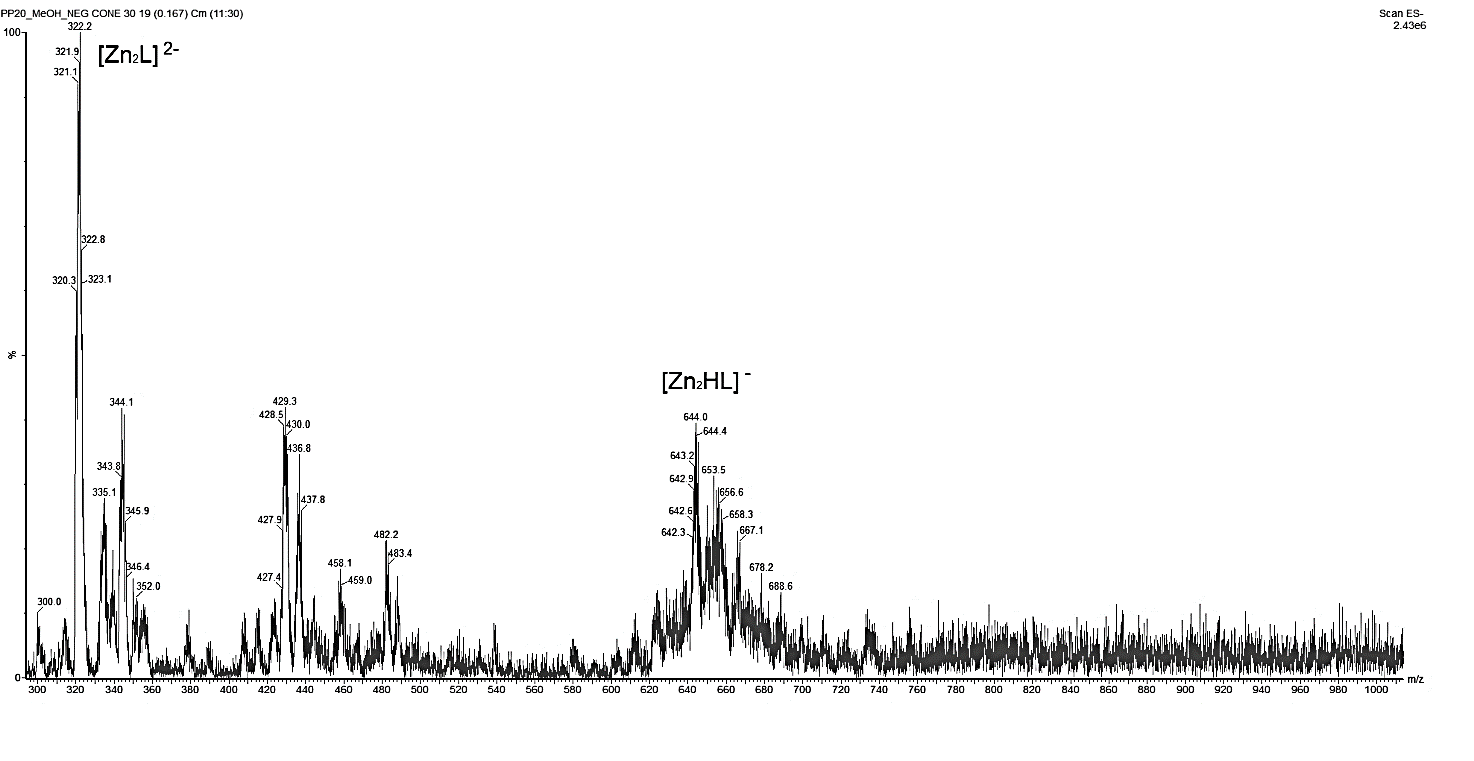

**Figure S15.** IR spectra of ligand **bis-CH** (Na_2_H_4_L^2^∙5H_2_O), complex **C2** (CuNaH_3_L^2^∙5H_2_O) and complex **C6** (Cu_2_NaHL^2^∙7H_2_O). The shift of the C=O stretching upon complexation indicates that in **C2** the ligand is coordinated in the keto form, while in **C6** it is in the enol form.


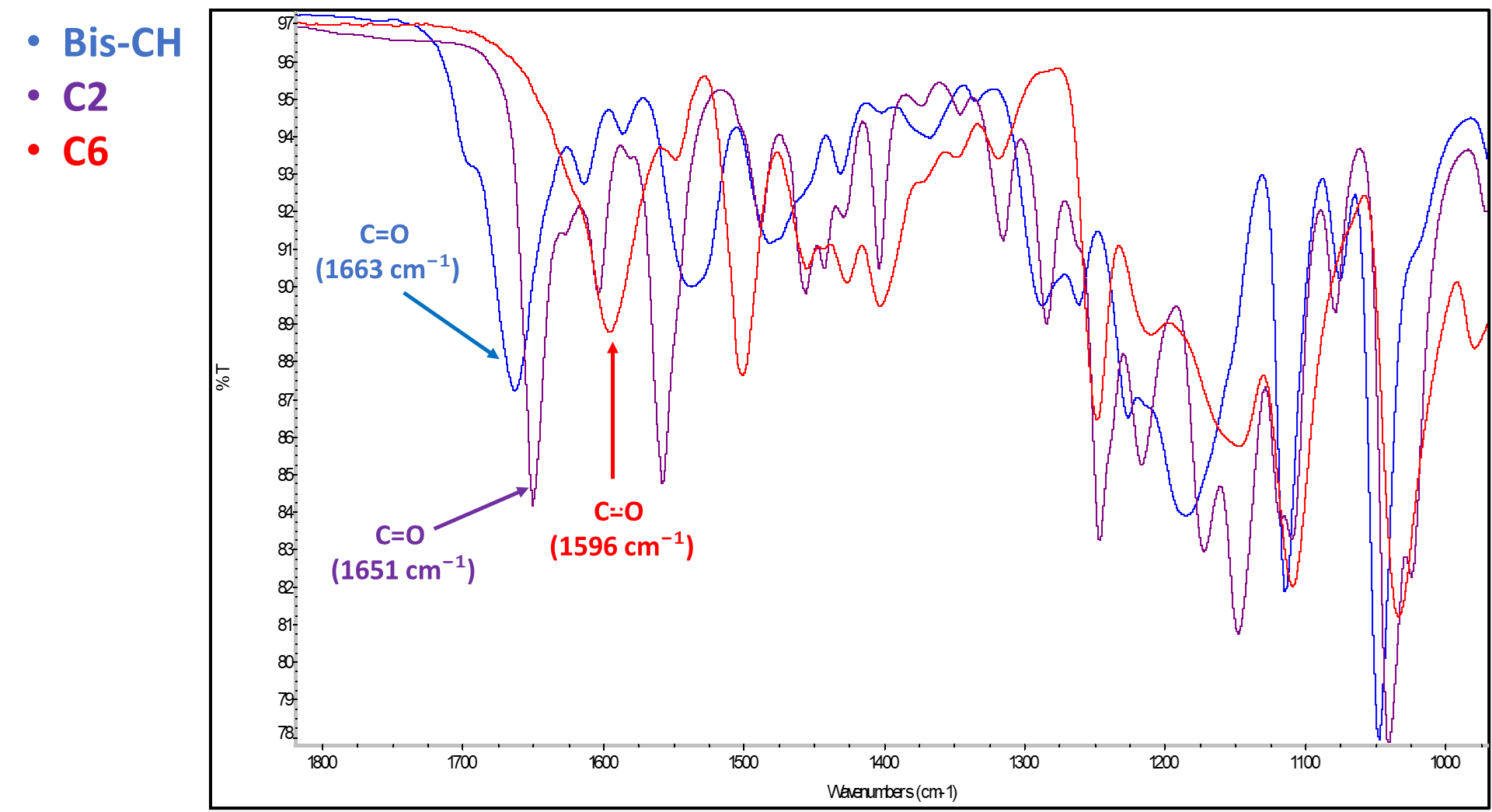


**Figure S16.** IR spectra of ligand **bis-CH** (Na_2_H_4_L^2^∙5H_2_O), and Zn(II) complexes **C4** and **C8**. The shift of the C=O stretching upon complexation indicates that the ligand is coordinated in the enol form in both complexes **C4** and **C8**.


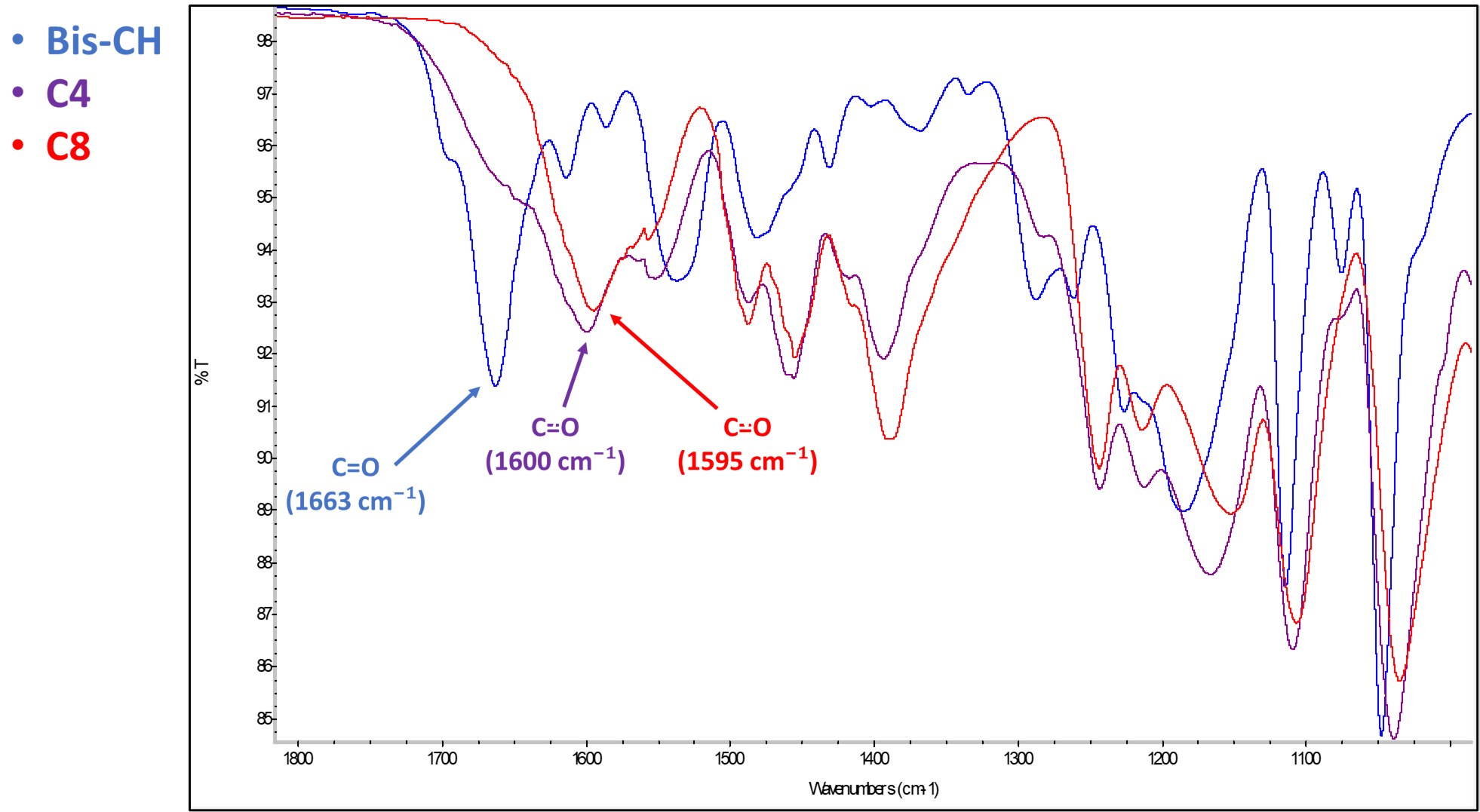


**Figure S17.** Zoom of the spectral region in which falls the isosbestic point at 288 nm for Na_2_H_4_L^1^·5.5 H_2_O. The red circles indicate the various positions of the isosbestic point at different pH values.

**Figure S18.** Zoom of the spectral region in which falls the isosbestic point at 284 nm for Na_2_H_4_L^2^·5 H_2_O. The red circles indicate the various positions of the isosbestic point at different pH values.


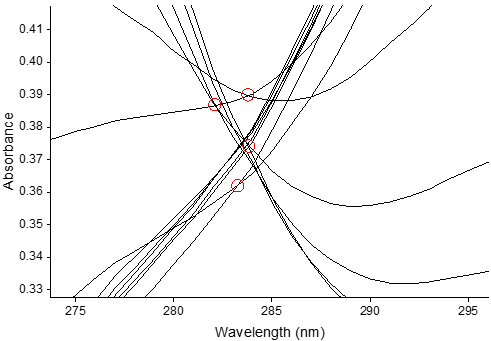


**Figure S19.** Representative distribution diagram of Na_2_H_4_L^1^·5.5 H_2_O and Cu(II) in aqueous solution.

**Figure S20.** UV-visible spectra for the titration of Na_2_H_4_L^1^·5.5 H_2_O with Cu(II) in aqueous solution at pH 7.4 (25 mM HEPES buffer; C_L_ = 17 µM; Cu(II):L = 0-0.8)

**Figure S21.** UV-visible spectra for the titration of Na_2_H_4_L^1^·5.5 H_2_O with Cu(II) in aqueous solution at pH 7.4 (25 mM HEPES buffer; C_L_ = 17 µM; Cu(II):L = 0.8-1.5)

**Figure S22.** Variation of the absorbance at 413 nm and pH 7.4 increasing metal to ligand ratio (ligand = **bis-TCH**).

**Fig. S23.** UV-visible spectra for the titration of Na_2_H_4_L^1^·5.5 H_2_O with Cu(II) in aqueous solution at pH 7.4 (25 mM HEPES buffer; C_L_ = 17 µM; Cu(II):L = 1.5-2).

**Figure S24.** Representative distribution diagram of **bis-CH** and Cu(II) in aqueous solution at pH 2.5

**Figure S25.** UV-visible spectra for the titration of Na_2_H_4_L^1^·5.5 H_2_O with Zn(II) in aqueous solution at pH 7.4 (25 mM HEPES buffer; C_L_ = 17 µM; Zn(II):L = 0-1).

**Figure S26.** UV-visible spectra for the titration of Na_2_H_4_L^1^·5.5 H_2_O with Zn(II) in aqueous solution at pH 7.4 (25 mM HEPES buffer; C_L_ = 17 µM; Zn(II):L = 1-2).

**Figure S27.** Diagram of percentage distribution of the species in solution with respect to the equivalents of Zn(II) added (ligand: **bis-TCH**).

**Figure S28.** Diagram of percentage distribution of the species in solution with respect to the equivalents of Zn(II) added (ligand: **bis-CH**).

**Figure S29.** Zoom on the square pyramidal coordination of one of the two Cu(II) ions in the crystal structure of [Cu_2_(NaHL^1^)(H_2_O)_7_]^.^3.5H_2_O.


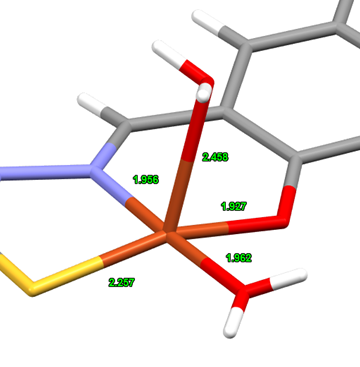


**Figure S30.** Zoom on the trigonal bipyramidal coordination of one of the two Cu(II) ions in the crystal structure of [Cu_2_(NaHL^1^)(H_2_O)_7_]^.^3.5H_2_O.


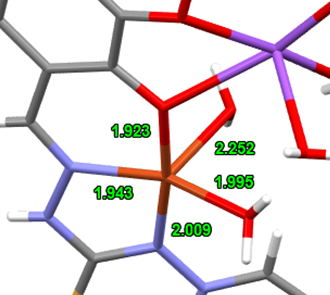


**Figure S31.** Zoom on the octahedral coordination of Na^+^ in the crystal structure of [Cu_2_(NaHL^1^)(H_2_O)_7_]^.^3.5H_2_O


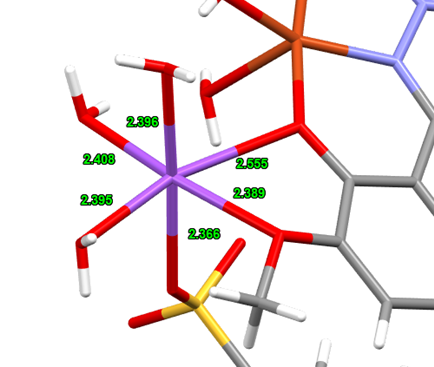


**Figure S32.** Dose–response curves obtained on Hs27 cells (left) and U937 cells (right) after 24-, 48- and 72 h treatment with the ligands Na_2_H_4_L^1^·5.5 H_2_O (A), Na_2_H_4_L^2^·5 H_2_O (B), and the Zn(II) complexes **C3** (C) and **C4** (D). Data are expressed as cell proliferation percentage compared with control cells.


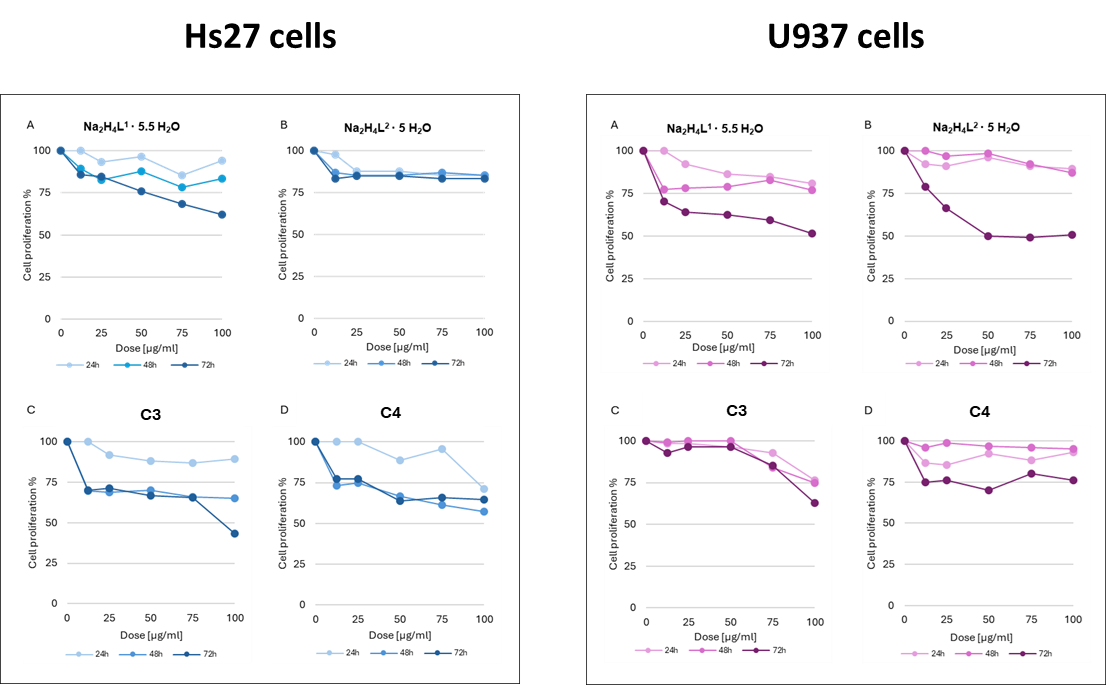


**Table S1.** GI_50_ values (µM/L) obtained in U937 and Hs27 cell lines after 24-, 48-, and 72 h treatment with the two ligands and the complexes **C1**-**C4**.

| Compound | GI_50_ value (µM/L) | | | | | |
| --- | --- | --- | --- | --- | --- | --- |
|  | **Hs27** | | | **U937** | | |
|  | 24 h | 48 h | 72 h | 24 h | 48 h | 72 h |
| bis-TCH | - | - | - | - | - | 144.30 ± 4.66 |
| bis-CH | - | - | - | - | - | 71.81 ± 6.80 |
| C1 | - | - | 69.50 ± 3.18 | 110.35 ± 4.73 | 120.00 ± 14.14 | 110.50 ± 2.82 |
| C2 | 107.75 ± 17.32 | 55.25 ± 7.42 | 53.00 ± 0.70 | 129.25 ± 0.35 | 77.00 ± 6.36 | 50.00 ± 14.14 |
| C3 | - | - | 117.75 ± 10.25 | - | - | - |
| C4 | - | - | - | - | - | - |
